# Divergence of the CYB5R3–mARC1 Redox Axis Across the Human MASLD-to-Hepatocellular-Carcinoma Continuum: An Exploratory Analysis of Liver-Biopsy and Tumor Cohorts

**DOI:** 10.3390/ijms27177806

**Published:** 2026-08-31

**Authors:** Soon Woo Nam

**Affiliations:** 1 Hepatobiliary Unit, Division of Gastroenterology, Department of Internal Medicine, Incheon St. Mary’s Hospital, Incheon 21431, Republic of Korea; drswnam@catholic.ac.kr; 2 College of Medicine, The Catholic University of Korea, Seoul 06591, Republic of Korea

**Keywords:** *CYB5R3*, *MTARC1*/mARC1, NAD^+^, reductive stress, de novo lipogenesis, *SCD*, hepatocellular carcinoma, MASLD, liver biopsy, exploratory analysis

## Abstract

CYB5R3 (NADH–cytochrome b5 reductase 3) and mARC1, the product of *MTARC1*, draw on the same cytochrome-b5 electron relay, so they are usually treated as two ends of one redox axis. Whether they behave as a unit across the continuum from metabolic dysfunction–associated steatotic liver disease (MASLD) to hepatocellular carcinoma (HCC) has not been established. In TCGA-LIHC, higher *CYB5R3* expression was associated with shorter overall survival in a continuous multivariable Cox model adjusted for age, sex, and stage (*n* = 339, 114 deaths; HR 1.23 per SD, 95% CI 1.01–1.48, *p* = 0.035), although it added essentially nothing to a clinical model containing age, sex, and stage (change in Harrell C-index 0.005, 95% CI −0.015 to +0.037). The direction was reproduced in the only independent cohort examined, GSE14520 (*n* = 242, 96 deaths; unadjusted HR 1.42 per SD, 95% CI 1.14–1.76, *p* = 0.0015), in which higher *MTARC1* was associated with longer survival (unadjusted HR 0.73 per SD, 95% CI 0.60–0.89, *p* = 0.0017); both single-gene estimates fall below conventional significance after adjustment for age, sex, and TNM stage (HR 1.24, 95% CI 0.99–1.56, *p* = 0.058 and HR 0.89, 95% CI 0.72–1.10, *p* = 0.29; *n* = 225, 86 deaths), although *CYB5R3* remains nominally significant in an adjusted model containing both genes (HR 1.27, 95% CI 1.01–1.59, *p* = 0.039), so that cohort replicates direction rather than independent prognostic value. Across the primary tumors of the analysis set, no correlation between the two transcripts was detectable (Spearman r_s_ = −0.03, 95% CI −0.13 to +0.08, *p* = 0.62; *n* = 339), so the axis is not demonstrably a unit at the expression level. In three human liver-biopsy cohorts spanning the MASLD histological spectrum (393 biopsies with fibrosis staging), *CYB5R3* gave estimates that differed in sign between cohorts and were uninformative when pooled (fibrosis stage, random-effects r_s_ = +0.01, 95% CI −0.24 to +0.26, q = 0.95, I^2^ = 83%). *MTARC1* behaved differently, falling with fibrosis stage in all three cohorts (pooled r_s_ = −0.22, 95% CI −0.32 to −0.13, q = 3 × 10^−5^, I^2^ = 0%), as did *PARP16* (−0.17, I^2^ = 0%), while *SCD* rose in all three (+0.13, I^2^ = 0%). This is consistent with the inconsistency being specific to *CYB5R3* rather than a property of the cohorts, although the fibrogenic positive controls were themselves heterogeneous (I^2^ = 53–62%), I^2^ is estimated from only three cohorts, and the difference between the *CYB5R3* and *MTARC1* coefficients was not formally tested. These observations are associative and hypothesis-generating. They provide no support for the widely assumed corollary that hepatic CYB5R3 becomes deficient as steatotic liver disease advances, so a therapeutic strategy predicated on that corollary currently rests on mouse gain-of-function data alone, without corroboration from human tissue. What they do support is narrower: within a single physically coupled electron-transfer axis, the two members behave differently before malignancy—*MTARC1* tracks fibrosis stage reproducibly while *CYB5R3* shows no reproducible relationship—and carry opposite prognostic directions after malignancy, significant for *MTARC1* only before covariate adjustment.

## 1. Introduction

Metabolic dysfunction–associated steatotic liver disease (MASLD) and its inflammatory form (MASH) [1] advance through fibrosis and cirrhosis toward hepatocellular carcinoma (HCC) along a continuum in which cellular redox balance is repeatedly implicated. Central to that balance is the NAD^+^/NADH couple: a surplus of NADH, termed reductive stress, promotes de novo lipogenesis, suppresses fatty-acid oxidation and fuels reactive oxygen species, and underlies common variation in human metabolic traits [2,3].

**Figure 1:**
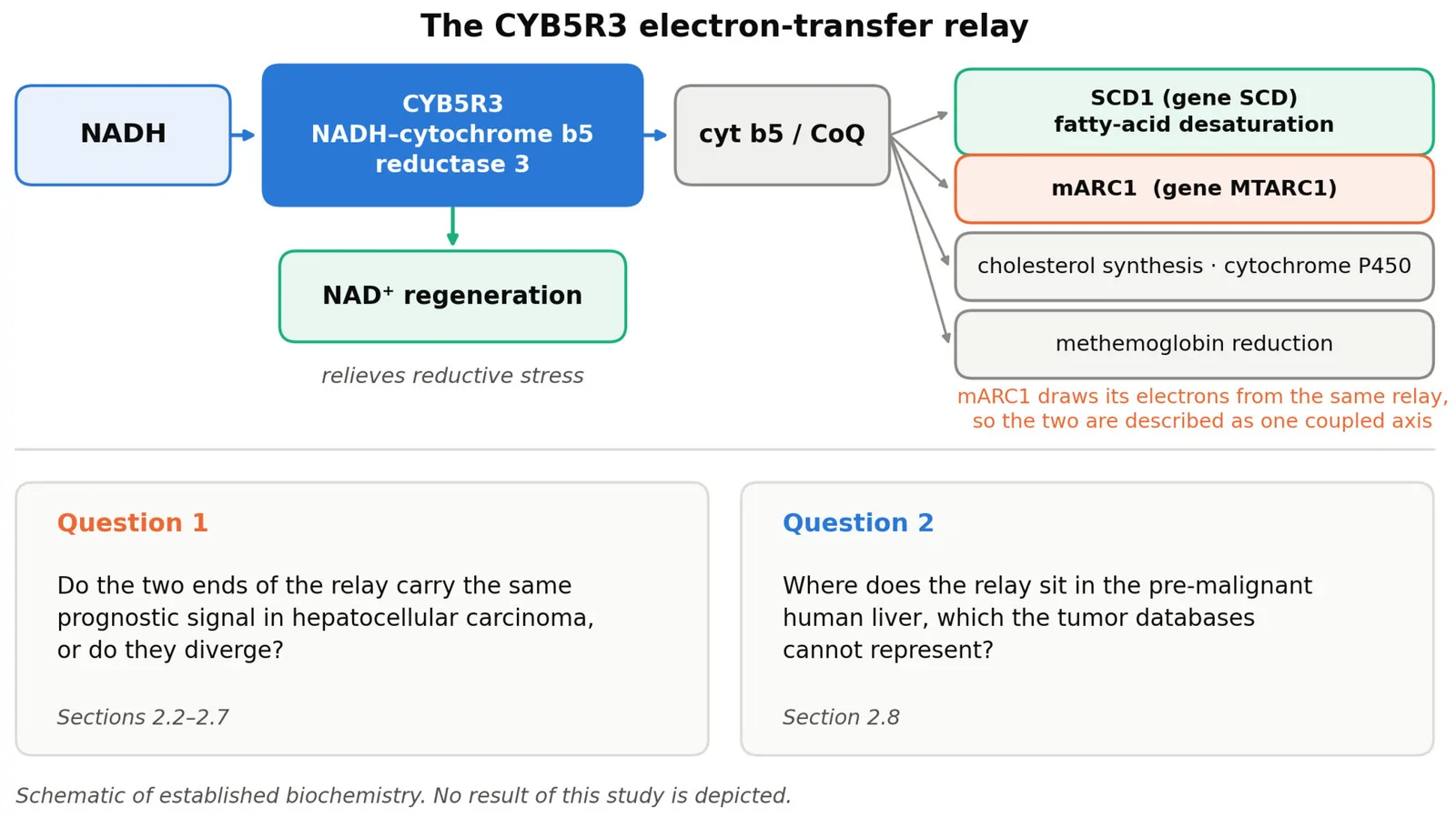
The CYB5R3 electron-transfer relay and the two questions this study asks of it. CYB5R3 oxidizes NADH to regenerate NAD^+^ and passes electrons through cytochrome b5 to several acceptors, among them SCD1 for fatty-acid desaturation, mARC1, cholesterol biosynthesis and cytochrome P450, and methemoglobin reduction. Because mARC1 draws its electrons from the same relay, the two proteins are usually described as one coupled axis; whether they behave as one is the first question (Section 2.2, Section 2.3, Section 2.4, Section 2.5, Section 2.6 and Section 2.7), and where the relay sits in the pre-malignant human liver is the second (Section 2.8). This diagram is a schematic of established biochemistry and does not depict a result of this study.

The relay and the two questions this study asks of it are summarized in Figure 1. CYB5R3 is a membrane-bound flavoenzyme that oxidizes NADH to regenerate NAD^+^ and relays electrons through cytochrome b5 and coenzyme Q to several acceptors, among them stearoyl-CoA desaturase (the protein SCD1, encoded by the gene *SCD*) for fatty-acid desaturation, cholesterol biosynthesis, cytochrome P450, methemoglobin reduction, and mARC1 [4,5]. Because mARC1 draws its electrons from the same cytochrome-b5-reductase relay, CYB5R3 and mARC1 are usually described as a single, physically coupled axis, and it is often assumed that the two behave as a unit. Co-overexpression of CYB5R3 with NQO1 reproduces features of caloric restriction and extends healthspan in mice [6,7], which has nominated the enzyme as a candidate metabolic-protective target. In a broad survey of tissues, CYB5R3 shows low tissue specificity and ubiquitous cytoplasmic and membrane expression across liver, adipose tissue, skeletal muscle, and vascular smooth muscle [8], so its output is likely to be shaped by the local metabolic setting rather than by presence or absence.

The oncological literature on CYB5R3 is internally inconsistent. In lung cancer, the enzyme behaves as a tumor suppressor: it is down-regulated in tumors, and its over-expression triggers endoplasmic-reticulum (ER)-stress apoptosis through the PERK–ATF4 and IRE1α–JNK pathways [9]. In estrogen-receptor–negative breast cancer, by contrast, high CYB5R3 promotes colonization and metastasis and marks poor survival [10]. Whether the liver resembles the lung or the breast has not been settled.

Two questions follow, and this study addresses them separately. First, do the two ends of the CYB5R3–mARC1 axis carry the same prognostic signal in HCC, or do they diverge? Second, where does the axis actually sit in the pre-malignant human liver, which the tumor databases cannot represent? The second question is the one the earlier literature has answered least directly. Mouse gain-of-function experiments have shown that raising CYB5R3 activity can be protective [6,7], and it is frequently inferred from this that endogenous CYB5R3 becomes insufficient as steatotic liver disease advances, so that reinforcing it would be therapeutic. That inference has not, to my knowledge, been tested against human liver histology. I therefore analyzed three public liver-biopsy cohorts with pathologist-assigned histological grades alongside the tumor cohorts.

This study is exploratory and hypothesis-generating. Its purpose is to establish, with explicit statistics and a released analysis pipeline, what can and cannot be claimed about this axis from human observational data, and to state the resulting model as a hypothesis rather than as a therapeutic program.

## 2. Results

### 2.1. Analytical Design: What Is Portal-Derived and What Was Analyzed Here

Public tumor portals and raw-data analysis answer different questions, and conflating them inflates apparent independence. I therefore separate the two throughout (Table 1). Portal-derived observations are the group comparisons and survival splits rendered by UALCAN [11], the optimal-cut-off survival estimates from KM Plotter [12], the CPTAC proteomic contrast [13], and the copy-number and alteration-frequency views of cBioPortal [14]. These are reported as portal outputs, with their own statistics, and are not treated as independent evidence.

Every claim in Section 2.2, Section 2.3, Section 2.4, Section 2.5, Section 2.6, Section 2.7, Section 2.8 and Section 2.9 that this study makes on its own account was regenerated from raw data by a scripted pipeline deposited in full (Data Availability), and no such value is read from a rendered plot. Portal figures are quoted alongside them at several points—the UALCAN row of Table 2, the UALCAN upper-quartile and KM Plotter optimal-cut-off estimates in Section 2.3, and the CPTAC and cBioPortal values in Section 2.5, the CPTAC value being restated in the legend of the corresponding figure in Section 2.5 and referred to qualitatively in the Discussion. Every one of these is collected in Table 3 or Table 1 and labeled as a portal output rather than treated as independent evidence. No result of this study is derived from any of these portal figures. The Human Protein Atlas description of tissue distribution in the Introduction is a background citation rather than a reported result, and is not tabulated for that reason. Two of the portal figures are approximate because the modules do not export them: the CPTAC protein medians and the cBioPortal alteration frequency. No value that this study reports as its own result is approximate.

Independence also needs to be stated plainly. UALCAN, cBioPortal, KM Plotter’s liver cohort, and the pan-cancer scan all draw on TCGA, so agreement among them measures agreement between different renderings of one dataset. Of the resources used here, only GSE14520 provides a genuinely independent survival cohort, and only the three liver-biopsy series provide data outside cancer altogether.

### 2.2. Tumor-Versus-Normal Expression, Regenerated from Raw Data, and Reconciliation of the Discordant Estimates

**Figure 2:**
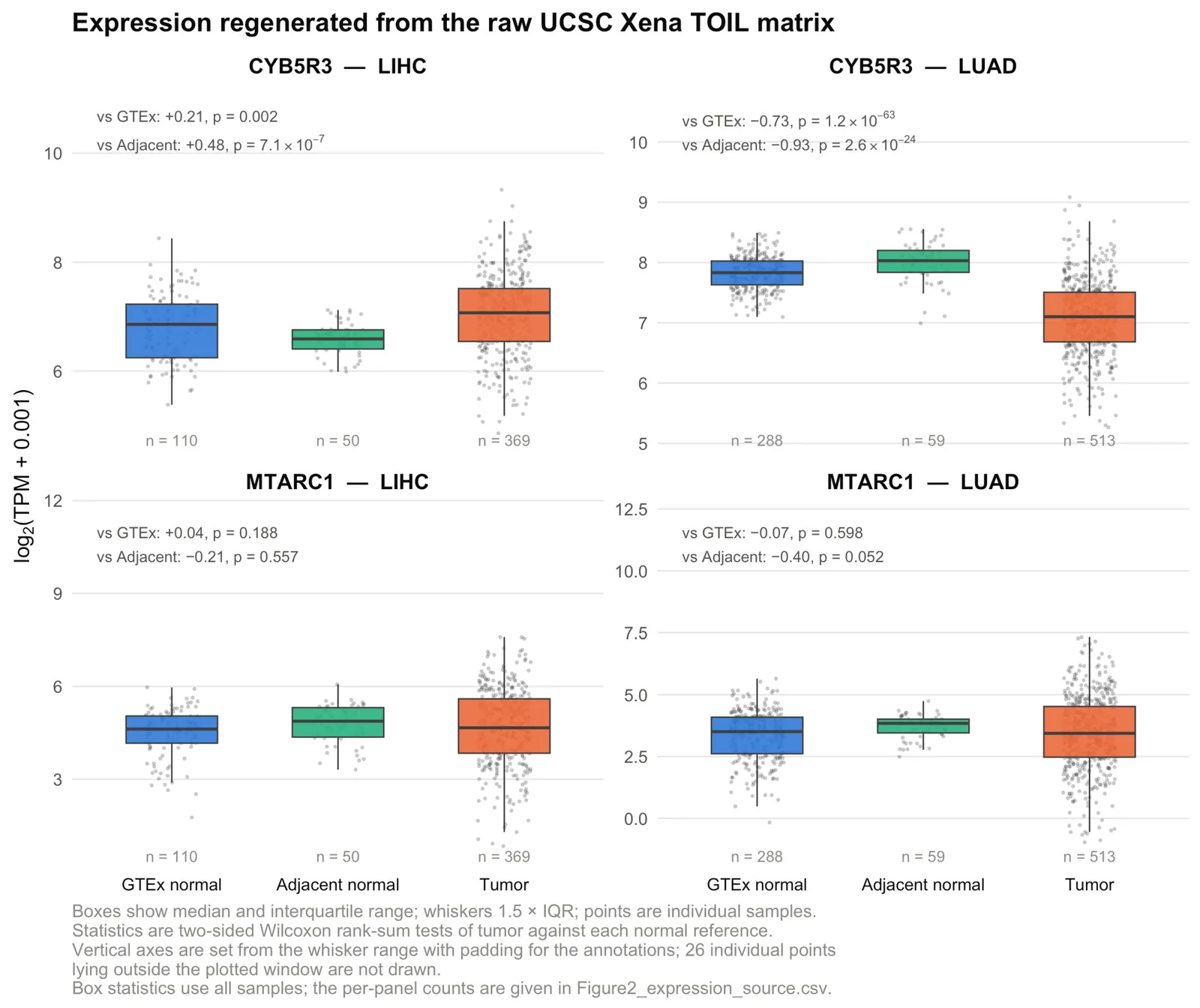
Tumor-versus-normal expression regenerated from raw data. Distributions of *CYB5R3* and *MTARC1* in TCGA-LIHC and TCGA-LUAD primary tumors against adjacent normal and against GTEx normal tissue, plotted from the Xena TOIL recompute, which is the only matrix carrying both genes and both normal references. Only the TOIL estimates are shown; for *CYB5R3* in liver, the two legacy-pipeline estimates point the opposite way (Table 2), which is why no over-expression claim is made. *MTARC1* estimates for both cohorts are in Supplementary Table S1. Boxes show median and interquartile range with *n* beneath each group; the log_2_ differences and Wilcoxon *p*-values against each normal reference are printed in each panel, in scientific e-notation below 1 × 10^−4^ and to three decimal places otherwise. Box statistics use every sample, but the vertical axes are set from the whisker range with padding added for the annotations, so a small number of individual points falling outside the plotted window are not drawn; their counts are given in the deposited source file. All values derive from the deposited pipeline; none is read from a rendered plot.

Regenerating the expression comparison from raw matrices produced the values in Table 2 and Figure 2. Two points require explicit comment, because the original submission made neither.

First, the direction of the *CYB5R3* tumor-versus-normal contrast in liver depends on which processing pipeline is used. The two estimates derived from the UCSC Xena TOIL recompute, in which TCGA and GTEx samples are processed through one uniform pipeline, both show higher expression in tumor: against TCGA adjacent normal, log_2_ fold change +0.48 (*p* = 7.1 × 10^−7^, Cliff’s δ = +0.43), and against the larger GTEx normal-liver reference, +0.21 (*p* = 0.0016, δ = +0.20). The two estimates derived from the legacy TCGA RNA-seq V2 pipeline both show a slightly lower, non-significant value in tumor: −0.07 on the HiSeqV2 matrix (*p* = 0.088, δ = −0.15) and −0.06 on the batch-corrected PanCanAtlas matrix (*p* = 0.102, δ = −0.14). On the linear scale, the reference estimate corresponds to a median of 134.5 TPM in tumor against 116.0 TPM in GTEx normal liver and 96.4 TPM in adjacent normal.

I pre-designated the TOIL contrast against GTEx normal liver as the reference estimate, because it uses a uniformly reprocessed matrix and the larger normal reference. Nevertheless, since the direction reverses with the pipeline rather than merely changing in magnitude, I do not claim that *CYB5R3* is over-expressed in HCC. Supplementary Table S1 reports all four estimates, and the caption of Supplementary Table S3 states that its liver estimate points the opposite way from the UALCAN rendering.

Second, the liver-versus-lung contrast does not depend on these choices. In LUAD, *CYB5R3* is substantially lower in tumor on every matrix and against every reference: −0.82 (*p* = 9.4 × 10^−25^) on HiSeqV2, −0.93 (*p* = 2.6 × 10^−24^) against adjacent normal and −0.73 (*p* = 1.2 × 10^−63^) against GTEx normal lung on the TOIL matrix, and −0.82 (*p* = 1.3 × 10^−24^) on the PanCanAtlas matrix, corresponding on the linear scale to 137.3 TPM in tumor against 227.8 TPM in GTEx normal lung. The tissue difference therefore rests on a robust reduction in lung set against a liver estimate that is, at most, a modest increase.

For *MTARC1*, the picture is similarly pipeline-dependent in liver (TOIL against GTEx normal, +0.04, *p* = 0.19; TOIL against adjacent normal, −0.21, *p* = 0.56; PanCanAtlas, −0.77, *p* = 2.1 × 10^−5^), and no claim about tumor-versus-normal direction is made for this gene either. *MTARC1* could not be retrieved from the legacy HiSeqV2 probe map under any of its aliases (*MTARC1*, *MARC1*, *MOSC1*), which is itself a reason to prefer the recompute; the failed lookup is reported to the console by the pipeline, and the absence of HiSeqV2 rows for *MTARC1* in results/10_expression_estimates.csv is its trace in the deposit.

### 2.3. Primary Prognostic Analysis in TCGA-LIHC

The continuous, per-standard-deviation multivariable Cox model is the primary prognostic analysis of this study. Median-split and optimal-cut-off estimates are reported as secondary, since a data-derived cut-off inflates apparent significance.

Of 423 TCGA-LIHC samples with *CYB5R3* expression, 371 were primary tumors with one sample retained per patient; 369 could be matched to *MTARC1* expression; 363 of those 369 had non-missing overall-survival time and status; and 339 had complete age, sex and AJCC stage. (A further two patients have survival data but no retrievable *MTARC1* value, so the serology subgroups in Section 2.9, which do not require *MTARC1*, begin from 365 patients with 130 deaths. The pan-cancer row in Section 2.7 also has 365 patients and 130 deaths, but reaches that number on the PanCanAtlas matrix by a different route and is not the same selection.) These 339 patients, among whom 114 died, constitute the primary analysis set (Supplementary Figure S6). Median follow-up by reverse Kaplan–Meier was 816 days (26.8 months). Stage was encoded as AJCC I–II versus III–IV, with stage IIIA–IIIC counted as III and IVA–IVB as IV. The 24 patients excluded for incomplete covariates were older (median 68.5 versus 61 years) and had a higher death rate (14 of 24 versus 114 of 339), but did not differ in *CYB5R3* expression (results/11_missing_compare.csv); a multiple-imputation sensitivity analysis using all 363 patients with both genes measured and survival data (m = 20, with the Nelson–Aalen cumulative hazard as an auxiliary variable) gave the same point estimate with slightly greater precision (*n* = 363, 128 deaths; HR 1.23 per SD, 95% CI 1.03–1.48, *p* = 0.026), so the exclusion of 24 patients does not create the association.

**Figure 3:**
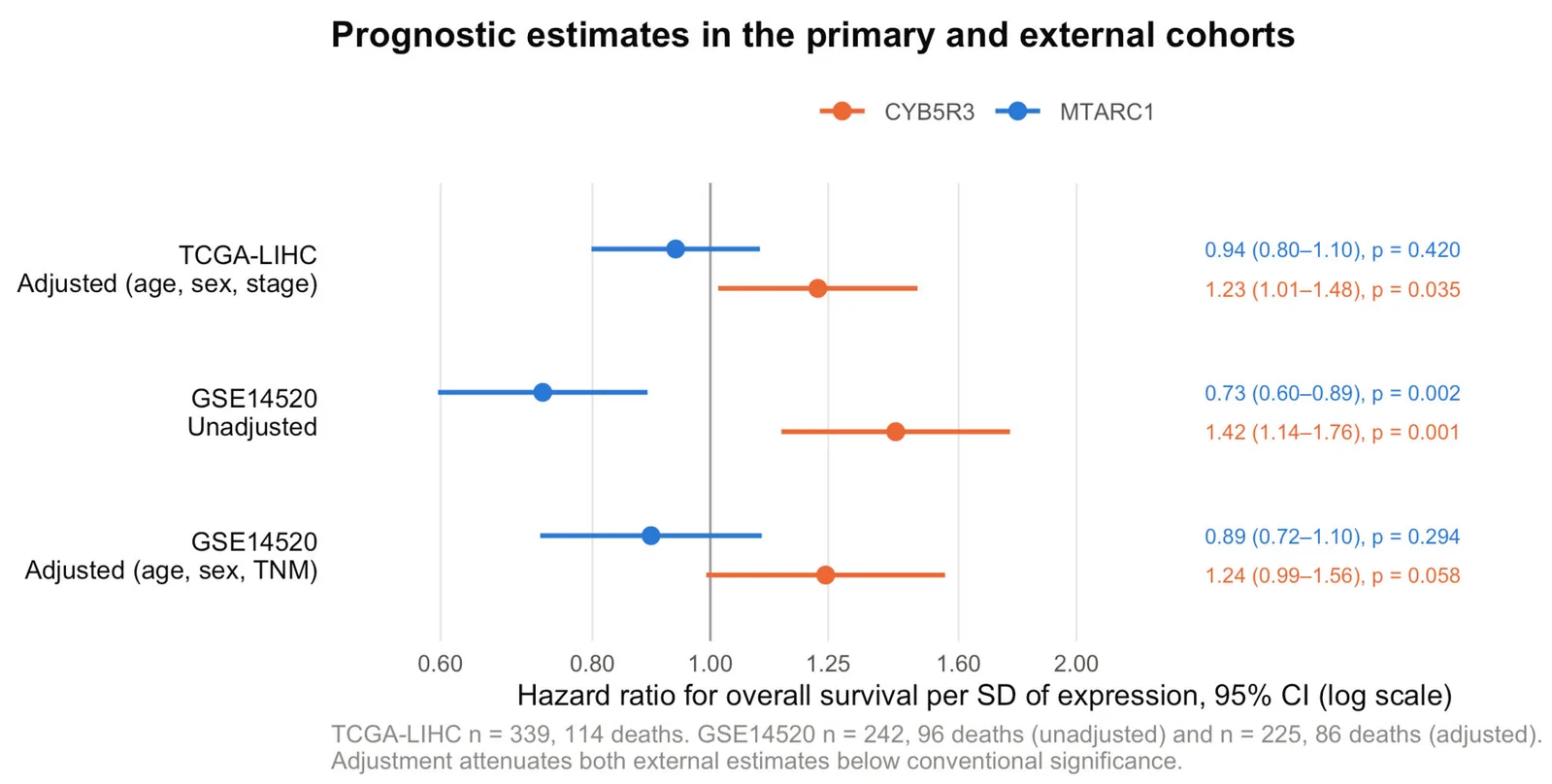
Primary and external prognostic estimates. Hazard ratios for overall survival per standard-deviation increase in expression, with 95% confidence intervals, for *CYB5R3* and *MTARC1* in three models: TCGA-LIHC adjusted for age, sex and stage (*n* = 339, 114 deaths); GSE14520 unadjusted (*n* = 242, 96 deaths); and GSE14520 adjusted for age, sex and TNM stage (*n* = 225, 86 deaths). Adjustment moves both external estimates below conventional significance.

Higher *CYB5R3* expression was independently associated with shorter overall survival: HR 1.23 per standard deviation, 95% CI 1.01–1.48, *p* = 0.035 (Figure 3). *MTARC1* showed the opposite direction but did not reach significance after adjustment within TCGA (HR 0.94 per SD, 95% CI 0.80–1.10, *p* = 0.42). Neither gene was associated with progression-free interval (162 events; *CYB5R3* HR 1.01 per SD, 95% CI 0.86–1.18, *p* = 0.93; *MTARC1* HR 1.02 per SD, 95% CI 0.88–1.17, *p* = 0.82), and both null results are reported here rather than omitted. A median split, which is secondary, gave HR 1.21 (95% CI 0.83–1.76, *p* = 0.33).

The complete set of survival models is given in Supplementary Table S2. Proportional hazards held. Scaled Schoenfeld residuals gave *p* = 0.91 for the *CYB5R3* term and a global *p* = 0.34; the residual plots for that model are Supplementary Figure S7. The corresponding values for the *MTARC1* model were *p* = 0.98 and *p* = 0.35, deposited in results/11_cox_ph.csv but not plotted. A penalized spline for expression did not improve on the linear term (likelihood-ratio χ^2^ = 1.41 on 2.05 degrees of freedom, *p* = 0.51; AIC 1134.8 linear versus 1137.5 spline), so the per-standard-deviation linear form is retained.

Discrimination is the question that determines whether this association is clinically meaningful, and the answer is largely negative. The Harrell C-index of a clinical base model containing age, sex, and stage was 0.629 (bootstrap 95% CI 0.580–0.688). Adding *CYB5R3* raised it to 0.634, a change of ΔC = 0.005 (95% CI −0.015 to +0.037, 1000 resamples). Adding *MTARC1* alone changed it by 0.001, and adding both by 0.008 (95% CI −0.010 to +0.050). On present evidence, *CYB5R3* expression adds essentially nothing to routine staging. For the same reason, the hazard ratio is not translated into an absolute-risk difference: with a ΔC-index whose confidence interval spans zero, an absolute-risk presentation would give the marker an appearance of clinical utility the discrimination data do not support.

The association also weakened when further clinical variables were added, although each addition reduced the analyzable sample. Adjusting additionally for log10 serum α-fetoprotein, floored at 1 ng/mL before the transformation, gave HR 1.16 (95% CI 0.92–1.47, *p* = 0.20; *n* = 261, 74 deaths); for vascular invasion, entered as present versus absent by collapsing the microvascular and macrovascular categories, HR 1.16 (95% CI 0.93–1.44, *p* = 0.20; *n* = 291, 85 deaths); for cirrhosis defined as Ishak stage 5–6, HR 1.17 (95% CI 0.89–1.55, *p* = 0.27; *n* = 196, 58 deaths); and for vascular invasion and cirrhosis together, HR 1.18 (95% CI 0.89–1.58, *p* = 0.25; *n* = 188, 54 deaths). The point estimates are consistent with the primary model, but the confidence intervals include unity, and I regard this as evidence that the association is not robust rather than as confirmation of it. Which clinical fields were available, and how complete each was, is given in results/11_extended_covariate_availability.csv; the α-fetoprotein, vascular-invasion, Ishak, and grade counts in that file are from the 339 patients in the primary analysis set, whereas the Child–Pugh count is from the full de-duplicated clinical matrix.

Because the two proteins are biochemically coupled, they were also modeled together. These joint analyses are exploratory: they were not pre-specified as primary endpoints, and *CYB5R3* is measured on the HiSeqV2 matrix while *MTARC1* is measured on the TOIL matrix, since *MTARC1* is absent from the legacy probe map (Section 2.2 and Section 4.5), so terms that combine the two genes combine two differently normalized pipelines. In a joint model, *CYB5R3* retained its estimate (HR 1.23, 95% CI 1.02–1.48, *p* = 0.032) and *MTARC1* did not become significant (HR 0.93, 95% CI 0.79–1.09, *p* = 0.38). A likelihood-ratio test of the joint model against the *CYB5R3*-only model was not significant (χ^2^ = 0.75, 1 df, *p* = 0.39), so *MTARC1* adds no detectable prognostic information beyond *CYB5R3* in this cohort, and a multiplicative interaction term added nothing either (HR 1.04, *p* = 0.57; χ^2^ = 0.32, *p* = 0.57). A single term expressing the imbalance between the two, the difference of the standardized expressions, was associated with survival (HR 1.14 per unit, 95% CI 1.01–1.29, *p* = 0.041), which is consistent with the two members contributing in opposite directions but is not independent evidence, since it is a linear combination of terms already tested.

Most informative of all, no correlation between the two transcripts was detectable across the 339 tumors of the analysis set (Spearman r_s_ = −0.03, 95% CI −0.13 to +0.08, *p* = 0.62), so any correlation between them is at most weak. Two proteins that share an electron relay need not share a transcript-level correlation, but this result means that the phrase “a single axis” cannot be justified from expression data. The manuscript no longer treats coordinated transcription as established; the two genes are still modeled jointly because their biochemical coupling motivates the comparison, but every joint, interaction, and difference term is labeled exploratory. It is the different behavior of the two members, not their coupling, that the data support.

For comparison, the portal-derived estimates were as follows: UALCAN upper-quartile split, high (above the upper quartile, *n* = 91) versus low/medium (*n* = 274), *p* = 0.001; KM Plotter with an optimally selected cut-off, HR 1.24, 95% CI 0.86–1.81, log-rank *p* = 0.25, which is not statistically significant. For *MTARC1*, KM Plotter gave HR 0.57, 95% CI 0.40–0.80, log-rank *p* = 0.0013. The confidence interval and *p*-value are reported wherever these hazard ratios appear, in the text and in Table 3.

### 2.4. External Replication with Covariate Adjustment

In GSE14520 [15] (HBV-related HCC, Affymetrix HG-U133A 2.0 and HT_HG-U133A; *n* = 242, 96 deaths), *CYB5R3* expression was associated with shorter survival (HR 1.42 per SD, 95% CI 1.14–1.76, *p* = 0.0015) and *MTARC1* with longer survival (HR 0.73 per SD, 95% CI 0.60–0.89, *p* = 0.0017) in unadjusted models of overall survival. Probe selection, preprocessing, and endpoint definition are now specified: the series matrices were used as deposited (RMA-normalized, log_2_), all probe sets annotated to the gene symbol were averaged, and the endpoint was overall survival in months with death from any cause as the event; probe identifiers are listed in results/11_gse14520_probe_selection.csv. The estimates above are unadjusted, and adjustment matters. In models restricted to the 225 patients with complete covariates (86 deaths), adjusting for age, sex, and TNM stage attenuated both associations below conventional significance: *CYB5R3* HR 1.24 per SD (95% CI 0.99–1.56, *p* = 0.058) and *MTARC1* HR 0.89 per SD (95% CI 0.72–1.10, *p* = 0.29). Adding cirrhosis and multinodularity changed little (*CYB5R3* HR 1.22, 95% CI 0.97–1.53, *p* = 0.089; *MTARC1* HR 0.88, 95% CI 0.71–1.09, *p* = 0.24). This cohort therefore replicates the direction of effect, not the claim of independent prognostic value, and the manuscript makes only the former claim.

One adjusted model does retain significance, and it is the one containing both genes rather than either gene alone. With both genes in the same adjusted model, *CYB5R3* remained adverse (HR 1.27 per SD, 95% CI 1.01–1.59, *p* = 0.039) while *MTARC1* did not reach significance (HR 0.87 per SD, 95% CI 0.70–1.07, *p* = 0.18), and the single term expressing the imbalance between them was associated with survival (HR 1.21 per unit, 95% CI 1.02–1.42, *p* = 0.025). The same pattern appears in TCGA-LIHC (Section 2.3). I note it because it is consistent across the two cohorts, not because it establishes anything: it is a linear combination of terms already tested, and it was not pre-specified as a primary endpoint.

### 2.5. Protein Level, Copy Number, and the Absence of a Genetic Dependency

**Figure 4:**
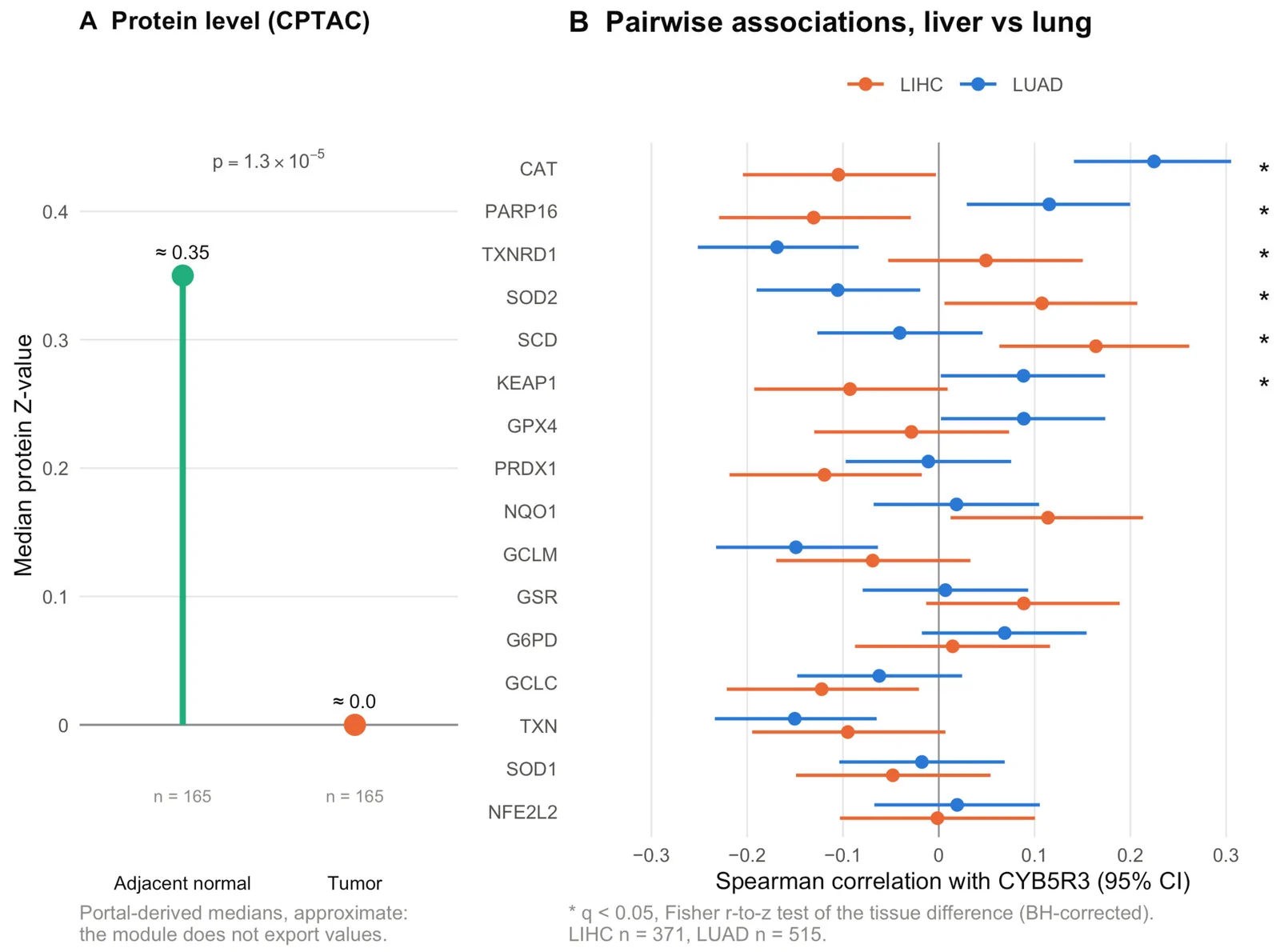
Protein abundance and pairwise expression associations. (**A**) CYB5R3 protein in HCC versus adjacent normal liver (CPTAC via UALCAN; *n* = 165/165; *p* = 1.3 × 10^−5^), lower in tumor. The two medians are approximate, marked with ≈ in the panel, because the module does not export values. Because the transcript direction is itself pipeline-dependent (Section 2.2), this panel is not presented as a transcript-versus-protein discordance. (**B**) Exploratory pairwise associations of *CYB5R3* with the pre-specified 16-gene redox panel in TCGA-LIHC (*n* = 371) and TCGA-LUAD (*n* = 515), showing Spearman coefficients with 95% confidence intervals, ordered by the q value of the liver-versus-lung difference; an asterisk marks q < 0.05 for that test. The coefficients, q-values, and the formal Fisher r-to-z test are in Table 4. Panel A is a portal-derived observation, and panel B is analyzed here.

Three observations complicate a simple reading, and all three are reported here rather than in the Discussion. Only the first of the three is shown in Figure 4A; the second has no figure, and the third is Supplementary Figure S1.

At the protein level (CPTAC via UALCAN), CYB5R3 was modestly lower in HCC tumors than in adjacent normal liver (median Z ≈ 0.0 versus ≈ +0.35; *n* = 165/165; *p* = 1.3 × 10^−5^). This agrees in direction with the two legacy-pipeline transcript estimates and disagrees with the two TOIL estimates (Table 2); since the transcript direction is itself pipeline-dependent (Section 2.2), no transcript-versus-protein discordance is claimed. Were the transcript in fact higher in tumor, a protein-level decrease would have more than one possible explanation. Differences in cohort, platform, tumor purity, and cellular composition between the transcriptomic and proteomic series can produce it. There is also a specific published mechanism: CYB5R3 is a substrate of the UFM1 system, and ufmylation at Lys214 inactivates the enzyme and directs it to lysosomal degradation through ER-phagy [16]. I note that mechanism as one candidate among several rather than as the explanation.

*CYB5R3* mRNA rose monotonically with copy-number status (shallow deletion < diploid < gain) in the cBioPortal rendering, and somatic *CYB5R3* or *SCD* alterations were rare (~1% of cases). Both are portal-derived descriptive observations; no relationship between copy number, mutation status, and outcome was tested here.

In DepMap CRISPR-knockout screens (Bioconductor depmap version 1.26.0; Supplementary Table S6, Supplementary Figure S1), *CYB5R3* is not a genetic dependency in either lineage, defined by DepMap lineage annotation rather than by the TCGA histology of Section 2.2 and Section 2.6, so the lung set is broader than LUAD: median Chronos gene effect −0.046 (IQR −0.078 to +0.002) in 22 liver lines and −0.018 (IQR −0.065 to +0.023) in 133 lung lines, with no liver line and one of the 133 lung lines below the −0.5 dependency threshold, and no significant difference between lineages (Wilcoxon *p* = 0.165) (results/15_depmap_dependency.csv). This *p*-value is produced by one deposited script and is the value used in the text, the supplement, and the figure caption. This is a negative result that constrains the interpretation, and it is stated as such in the Conclusions rather than mentioned only in passing.

### 2.6. Exploratory Pairwise Associations of CYB5R3 in Liver Versus Lung

I examined a pre-specified 16-gene redox panel, which includes *SCD* and *PARP16*, in primary tumors of TCGA-LIHC and TCGA-LUAD (Figure 4B; complete panel in Supplementary Table S5 and Supplementary Figure S5). These are exploratory pairwise associations, not a co-expression module, and they are described as such throughout; the earlier term “module” overstated what a small set of pre-selected genes with weak correlations can support.

The full panel is reported in Table 4 with coefficients and Benjamini–Hochberg q-values for both tissues and the formal test of the tissue difference, including *CAT* and *SOD2*, which previously appeared without q-values; the 95% confidence interval and *p*-value of every coefficient are in Supplementary Table S5 and in results/12_coexpression_full.csv. In TCGA-LIHC (*n* = 371), only *SCD* survives FDR correction (r_s_ = +0.164, 95% CI +0.063 to +0.261, q = 0.024). The associations the original submission emphasized do not: *PARP16* r_s_ = −0.131 (q = 0.086) and *NQO1* r_s_ = +0.114 (q = 0.090). In TCGA-LUAD (*n* = 515) six genes survive correction: *CAT* (+0.225, q = 4 × 10^−6^), *TXNRD1* (−0.169, q = 0.001), *TXN* (−0.150, q = 0.003), *GCLM* (−0.149, q = 0.003), *PARP16* (+0.115, q = 0.028) and *SOD2* (−0.106, q = 0.044). Coefficients of this size account for roughly 1–5% of between-patient variance, and no mechanistic conclusion follows from any of them alone.

Three analyses that the original submission lacked now test the tissue contrast directly, and they do not all point the same way.

Counting sign reversals is uninformative. Eight of the sixteen panel genes reverse sign between liver and lung. Permuting *CYB5R3* expression within each cohort 10,000 times produced a mean of 8.0 reversals (SD 2.3; 95th percentile 12), so the observed count is exactly what chance yields (permutation *p* = 0.58). Any argument resting on the number of apparent reversals is therefore unsupported, and the original manuscript’s appeal to a general sign-flip pattern has been removed.

Testing specific coefficients is informative. Comparing the liver and lung coefficients gene by gene with Fisher’s r-to-z transformation, six differ significantly after Benjamini–Hochberg correction across the panel: *CAT* (−0.105 versus +0.225; z = −4.88, q = 1.7 × 10^−5^), *PARP16* (−0.131 versus +0.115; z = −3.62, q = 0.0024), *TXNRD1* (+0.049 versus −0.169; z = 3.22, q = 0.0069), *SOD2* (+0.108 versus −0.106; z = 3.13, q = 0.0070), *SCD* (+0.164 versus −0.041; z = 3.02, q = 0.0081) and *KEAP1* (−0.093 versus +0.088; z = −2.66, q = 0.021). Of the remaining ten, two (*GPX4* and *NFE2L2*) reverse sign, but the difference does not survive correction, and eight show no significant tissue difference at all.

The six significant differences survive adjustment for tumor cellular composition, which is a recognized cause of exactly this kind of spurious reversal. Conditioning on a marker-based proxy for tumor content (Section 4.6), all six remain significant after correction (*CAT* q = 1.3 × 10^−4^, *PARP16* q = 0.0018, *SOD2* q = 0.0018, *SCD* q = 0.0052, *TXNRD1* q = 0.0091, *KEAP1* q = 0.024). The liver *SCD* association strengthens rather than weakens under adjustment (adjusted r_s_ = +0.195, q = 0.003), whereas the liver *PARP16* association weakens (adjusted r_s_ = −0.102, q = 0.26). Full raw and adjusted coefficients are in results/12_purity_adjusted.csv and results/12_tissue_difference_purity_adjusted.csv.

The correct summary is therefore narrower than the one the original submission offered. The two genes on which the tissue-context hypothesis turns, *SCD* and *PARP16*, do differ significantly between liver and lung in the direction the model predicts, and that difference survives adjustment for the composition proxy, which is a weaker control than a purity call (Section 4.6). But the coefficients are small, only one survives correction within liver, and the broader claim of a tissue-inverted redox program is not supported. Where the formal test does not survive correction, the observation is described as consistent with the tissue-context hypothesis rather than as evidence for it.

### 2.7. Pan-Cancer Scan with Multiplicity Control

Across TCGA cohorts with at least 20 evaluable tumors and 10 deaths, *CYB5R3* expression was related to overall survival by univariable Cox regression, and tumor-versus-normal expression was compared where at least 10 adjacent-normal samples existed. Twenty-eight cohorts met the survival criteria, and 15 of those also had at least 10 adjacent-normal samples for the expression comparison. The complete table, with Benjamini–Hochberg correction applied across cohorts within each of the two families separately, is provided in Supplementary Table S3; its survival family is plotted in Supplementary Figure S2, and the two families are compared in Supplementary Figure S3. The complete table is deposited as results/13_pancancer_full.csv.

After correction for multiplicity across cohorts, no cohort retains a significant association between *CYB5R3* expression and overall survival, including LIHC. The nominal LIHC estimate (HR 1.20 per SD, 95% CI 1.01–1.44, *p* = 0.044) becomes q = 0.19, and the smallest corrected value anywhere in the survival family is q = 0.146, shared by HNSC, KIRC, MESO and SKCM, all nominally adverse. The alternative, leaving the scan uncorrected and saying so, would have produced a longer list of nominally significant cohorts; correction leaves none, and that is what is reported.

Two clarifications are made in the output itself. The LIHC row draws on the same cohort as the primary analysis in Section 2.3—it is the univariable estimate in the 365 patients with survival data on the PanCanAtlas matrix, of whom 339 have the complete covariates the primary model requires; that set of 365 is the same size as the one used in Section 2.9 but is reached by a different route and is not the same selection—and is therefore flagged as such; it is not an independent reproduction, and the earlier description of it as a reproduction has been removed. Across the broader panel, the simple “up-and-adverse, down-and-protective” rule is not supported (Supplementary Figure S3; Spearman r_s_ = 0.26, *p* = 0.35 across 15 cohorts, which at that number is an absence of evidence rather than evidence of absence), so the liver–lung difference is presented as a two-tissue observation and not as a general law.

### 2.8. The Axis Across the Human MASLD Histological Spectrum

**Figure 5:**
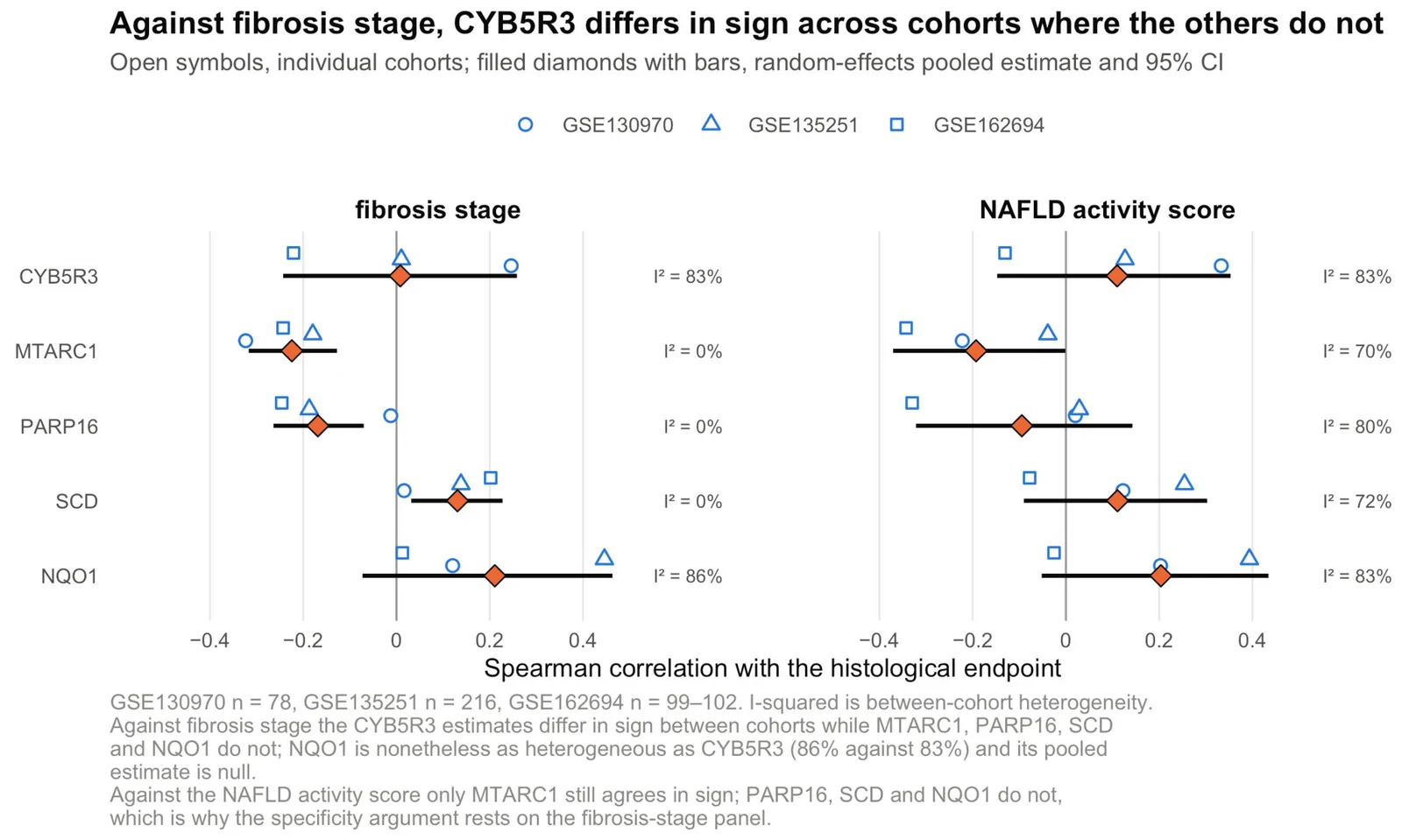
The CYB5R3–mARC1 axis across the human MASLD spectrum. Spearman coefficients relating *CYB5R3*, *MTARC1*, *PARP16*, *SCD* and *NQO1* to pathologist-assigned fibrosis stage (left panel) and NAFLD activity score (right panel) in the three liver-biopsy cohorts. Open symbols are the individual cohorts; filled diamonds with bars are the random-effects pooled estimate and its 95% confidence interval, with I^2^ printed alongside. The three cohort estimates for *CYB5R3* against fibrosis stage differ in sign (+0.246, +0.010 and −0.221; pooled r_s_ = +0.008, I^2^ = 83%), so no relationship in either direction is established. *MTARC1* falls with fibrosis stage in all three cohorts (pooled r_s_ = −0.224, I^2^ = 0%), as does *PARP16* (−0.169, I^2^ = 0%), while *SCD* rises consistently (+0.131, I^2^ = 0%). *NQO1* also agrees in sign against fibrosis stage, but its pooled estimate is as heterogeneous as that of *CYB5R3* (I^2^ = 86% against 83%) and is null, so no pooled claim is made for it. Against the NAFLD activity score, only *MTARC1* still agrees in sign across all three cohorts, while *PARP16*, *SCD* and *NQO1* do not, which is why the specificity argument rests on the fibrosis-stage panel. Cohort-level estimates are shown rather than averaged away.

The tumor cohorts cannot represent the pre-malignant metabolic liver, which was the principal limitation of the original submission. I therefore analyzed three public liver-biopsy series with pathologist-assigned histology: GSE130970 (78 biopsies) [17], GSE135251 (216 biopsies) [18] and GSE162694 (128 of 143 biopsies could be matched to their annotation; 99 with fibrosis stage and 102 with NAFLD activity score) [19]. In total, 393 biopsies contributed a fibrosis-stage estimate and 396 an activity-score estimate. I tested *CYB5R3*, *MTARC1*, *SCD* and *NQO1*, with *PARP16* and *FASN* as mechanistically adjacent genes and *COL1A1* and *TGFB1* as fibrogenic positive controls, applying Benjamini–Hochberg correction across all 72 gene × histology × cohort tests and pooling by random-effects meta-analysis of Fisher-z-transformed coefficients with I^2^ reported. Figure 5 shows the fibrosis-stage and activity-score estimates for *CYB5R3*, *MTARC1*, *PARP16*, *SCD* and *NQO1*; Table 5 adds *FASN*, the two fibrogenic controls, and the three single-cohort lesion grades for *CYB5R3* and *MTARC1*, and the complete gene × histology × cohort output is Supplementary Table S7.

The central result is that *CYB5R3* behaves inconsistently, and that *MTARC1*, *PARP16*, and *SCD* do not. The three cohorts do not agree even on the sign of its relationship to fibrosis stage: +0.246 in GSE130970 (q = 0.068), +0.010 in GSE135251 (q = 0.93) and −0.221 in GSE162694 (q = 0.065). Pooled, the estimate is indistinguishable from zero with extreme heterogeneity (r_s_ = +0.008, 95% CI −0.243 to +0.258, q = 0.95, I^2^ = 83%). The same is true against the NAFLD activity score (+0.333, +0.127, and −0.131 by cohort; pooled +0.110, 95% CI −0.147 to +0.353, q = 0.50, I^2^ = 83%).

This cannot be attributed to platform or scoring differences alone, because three genes measured in the same biopsies, on the same platform, and against the same pathologist-assigned scores within each cohort, give consistent estimates. *MTARC1* falls with fibrosis stage in all three cohorts (−0.323, −0.180, and −0.243; pooled r_s_ = −0.224, 95% CI −0.317 to −0.128, q = 3 × 10^−5^, I^2^ = 0%). *PARP16*, the NAD^+^-dependent ER-resident mono-ADP-ribosyltransferase on which the tissue-context hypothesis turns, is negative in all three cohorts and significantly so in two, with a null estimate in GSE130970 (r_s_ = −0.012, q = 0.93); pooled, it is −0.169 (95% CI −0.264 to −0.070, q = 0.002, I^2^ = 0%). *SCD*, the desaturase to which CYB5R3 donates electrons, is positive in all three and significant in none individually (GSE130970 +0.016, q = 0.93); pooled, it is +0.131 (95% CI +0.032 to +0.228, q = 0.023, I^2^ = 0%). These are agreements in sign with a pooled estimate that clears correction, not three independent replications. Against fibrosis stage, *CYB5R3* and *NQO1* are the only transcripts whose pooled estimate is both heterogeneous and null; the fibrogenic controls *COL1A1* and *TGFB1* are somewhat heterogeneous too (I^2^ = 62% and 53%) but agree in sign across all three cohorts. Against the NAFLD activity score, all four of *MTARC1*, *PARP16*, *SCD*, and *CYB5R3* are heterogeneous (I^2^ 70–83%), so the specificity argument rests on the fibrosis-stage comparison.

Two conclusions follow, and the first is the one that matters for the therapeutic question the original submission raised. The premise that hepatic *CYB5R3* becomes deficient as steatotic liver disease advances is not supported by human liver histology. It is not refuted either: the cohorts disagree in direction, so the relationship is simply not established. A therapeutic strategy predicated on that premise therefore currently rests on mouse gain-of-function data alone and has no corroboration from human tissue. This is a weaker statement than the one I made in the original submission and a weaker statement than a single cohort would have supported, and it is the one the data license.

The second is that the two members of the axis behave differently in the pre-malignant liver, and that this difference is now the better-supported half of the finding. *MTARC1* tracks fibrosis reproducibly; *CYB5R3* tracks nothing reproducibly. The contrast between the two coefficients was not itself tested: the evidence is that one pooled estimate clears correction and the other does not, and the confidence interval for *CYB5R3* (−0.243 to +0.258) contains the *MTARC1* point estimate, so the difference between them is a difference in what each is individually consistent with rather than a demonstrated difference between the two. In GSE130970, where the individual MASH lesions are graded separately, the dissociation is visible within a single cohort as well: in a linear model containing steatosis, lobular inflammation, and ballooning simultaneously, lobular inflammation was the only significant term for *MTARC1* (β = −0.240, *p* = 0.0036, q = 0.038) whereas for *CYB5R3* only ballooning retained an independent association, and it does not survive correction (β = 0.081, *p* = 0.029, q = 0.14), while lobular inflammation contributed nothing (β = 0.042, *p* = 0.48, q = 0.62). Partial Spearman correlations adjusting for the other two lesions gave the same picture (*MTARC1* against lobular inflammation, partial r_s_ = −0.32, *p* = 0.004, q = 0.038; *CYB5R3* against ballooning, +0.27, *p* = 0.018, q = 0.097; *CYB5R3* against lobular inflammation, −0.02, *p* = 0.86, q = 0.88). The two *CYB5R3*–lobular-inflammation estimates, the model coefficient and the partial correlation, differ in sign but are both indistinguishable from zero.

### 2.9. Stage, Grade and Etiology Within TCGA-LIHC

These analyses appeared in the Discussion of the original submission and are moved here, with their methods in Section 4, the full output in Supplementary Table S4, and the group medians in Supplementary Figure S4. They have been regenerated from raw data by the deposited pipeline; the values differ slightly from those reported in the original submission, and the regenerated values are the ones given here.

*CYB5R3* expression showed no monotone trend across AJCC stage (stage I *n* = 171, II *n* = 86, III *n* = 85, IV *n* = 5; Jonckheere–Terpstra *p* = 0.164) but showed a significant decreasing trend across histologic grade (G1 *n* = 55, median 12.84; G2 *n* = 177, 12.85; G3 *n* = 122, 12.71; G4 *n* = 12, 12.37; Jonckheere–Terpstra *p* = 0.007), although the group medians are not strictly monotone and the highest-grade group contains only 12 patients. A gene whose expression falls as tumors become less differentiated, while its high expression marks worse survival, is not straightforwardly a tumor-promoting gene, and this tension is carried into the Limitations and the Conclusions rather than reported once in passing.

No metabolic subset can be defined from these data, and the analysis reported in the original submission rested on a misreading of the field. The TCGA-LIHC viral_hepatitis_serology field lists the serologies that returned a positive result; it is populated for 164 of 371 primary tumors. An empty field therefore means that no positive serology was recorded, which cannot be distinguished from a patient who was never tested. It is not equivalent to non-viral etiology, still less to metabolic liver disease. The group described in the original submission as the “non-viral/metabolic subset” was constructed on the opposite reading of this field, and the estimate derived from it (HR 1.46, 95% CI 0.58–3.68, *p* = 0.42, *n* = 18) is withdrawn.

What can be reported is the comparison as the field actually supports it. Among the 371 primary tumors, 164 have at least one positive viral serology recorded, and 207 do not, and *CYB5R3* expression does not differ between them (median 12.82 versus 12.78, Wilcoxon *p* = 0.209). In exploratory univariable survival models—unadjusted, unlike every other hazard ratio in this section—the adverse direction is present in both groups and is numerically larger in the group without a recorded positive serology, which contains an unknown mixture of non-viral cases and patients who were never tested: HR 1.30 per SD (95% CI 0.98–1.71, *p* = 0.064; *n* = 203, 59 deaths) versus HR 1.13 per SD (95% CI 0.88–1.45, *p* = 0.34; *n* = 162, 71 deaths) in the viral-serology-positive group. Neither estimate is significant, neither group is metabolically defined, and no formal comparison between them is made.

The consequence is that this study contains no survival estimate in a metabolically defined population. That is a more consequential limitation than the underpowered subgroup estimate it replaces, and it is stated as such in Section 3.5.

## 3. Discussion

### 3.1. What These Data Support

Three findings survive the analyses above. Within HCC, higher *CYB5R3* expression is associated with shorter overall survival, independently of age, sex, and stage, and the direction replicates in the one independent cohort available. In TCGA-LIHC, the *MTARC1* estimate points the opposite way but is not significant (HR 0.94, *p* = 0.42); in GSE14520, the opposite direction is significant before adjustment but not after it. In the pre-malignant liver, *MTARC1* falls with fibrosis stage reproducibly across three independent biopsy cohorts with no detectable heterogeneity, whereas *CYB5R3* gives estimates that differ in sign between the same three cohorts.

More findings do not survive, and reporting them is the substance of this revision. *CYB5R3* is not reliably over-expressed in liver tumors: the direction reverses with the processing pipeline. Its protein is not more abundant in tumors than in normal liver. Its expression shows a decreasing trend, rather than an increasing one, across histologic grade. It is not a genetic dependency in liver cancer cell lines. Its correlations with the redox panel are weak, and only *SCD* survives correction on the liver side. The number of apparent liver-versus-lung sign reversals is exactly what permutation yields. It is not associated with progression-free interval. In the external cohort, the single-gene associations fall below conventional significance once age, sex, and stage are adjusted for. Across 28 TCGA cohorts, nothing survives correction for multiplicity, including LIHC. And, most consequentially for any clinical reading, it adds essentially nothing to a model containing age, sex, and stage (ΔC-index 0.005, 95% CI −0.015 to +0.037).

One further result bears directly on the framing of the whole study. No correlation between the two transcripts was detectable across the primary tumors of the analysis set (r_s_ = −0.03, 95% CI −0.13 to +0.08, *p* = 0.62; *n* = 339), and in a joint model, *MTARC1* added no detectable prognostic information beyond *CYB5R3* (likelihood-ratio *p* = 0.39). Whatever coupling exists between CYB5R3 and mARC1 at the level of electron transfer, it does not produce a detectable coordinated transcriptional signal, and the manuscript no longer treats “one axis” as an inferential premise, although the two genes are still modeled jointly as an exploratory comparison. Taken together, these results describe a modest prognostic gradient in a pair of genes that behave differently, not a tumor dependency and not a druggable node.

### 3.2. A Tissue-Context Hypothesis, Stated as a Hypothesis

**Figure 6:**
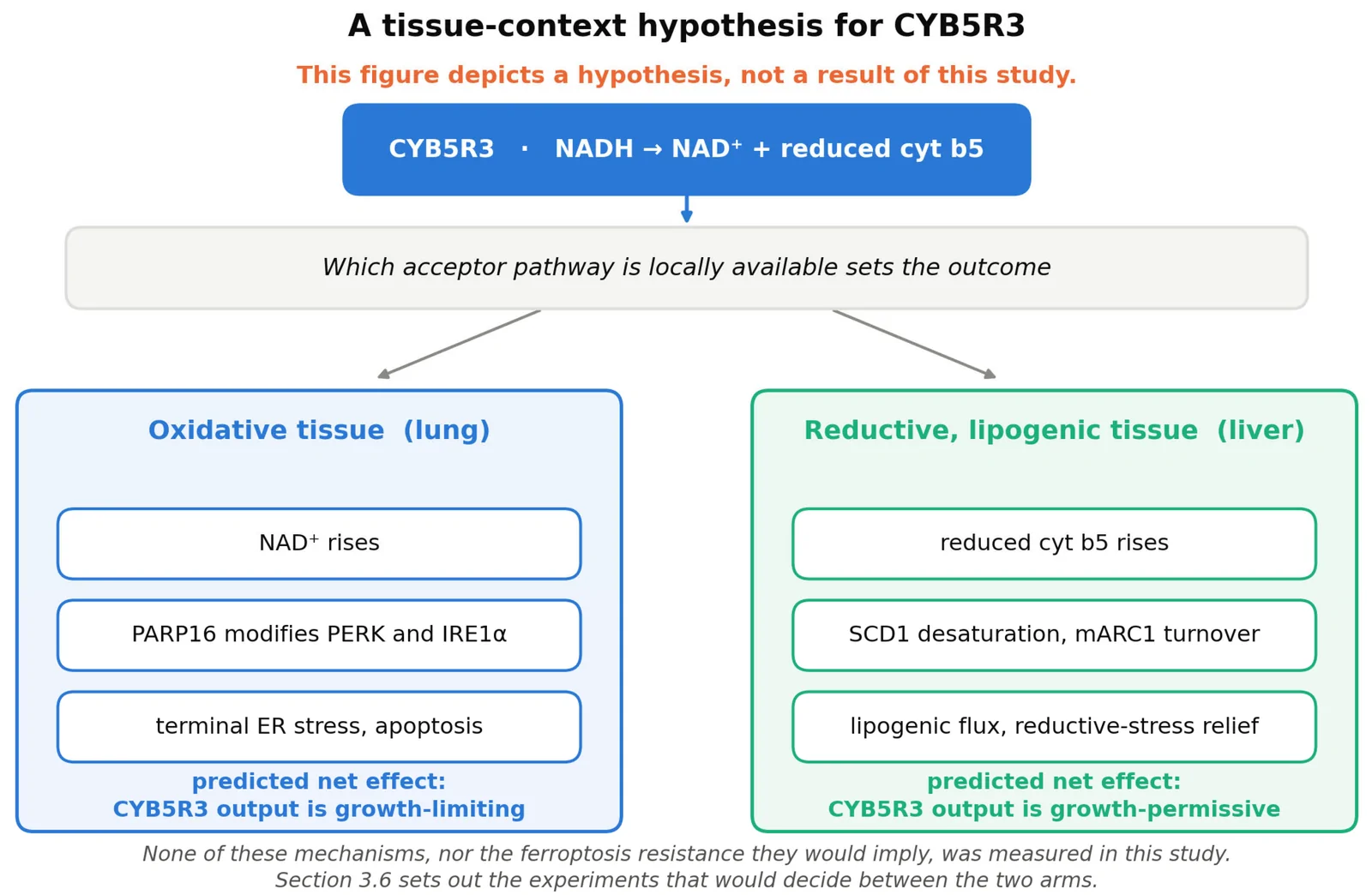
Tissue-context hypothesis. A schematic of the proposed dependence of CYB5R3’s downstream effect on locally available acceptor pathways, with ER-stress-associated death in the oxidative lung and lipogenic desaturation in the reductive liver. This figure depicts a hypothesis, not a result. No experiment reported here measures electron routing, ferroptosis, or ER stress.

The observation that motivated this study, that CYB5R3 suppresses tumors in lung yet marks poor prognosis in liver, still requires an explanation, and the most economical one is that CYB5R3 is neither pro- nor anti-tumor in itself but an electron and NAD^+^ dispenser whose downstream fate is set by the metabolic wiring of the host tissue (Figure 6). In lung cancer cells, CYB5R3 overexpression raises NAD^+^, which activates the ER-resident mono-ADP-ribosyltransferase PARP16 to modify PERK and IRE1α, driving terminal ER stress and apoptosis [9]. In hepatocytes, which are the body’s lipogenic and NAD^+^-central hub, the same electron output feeds SCD1 and the generation of monounsaturated fatty acids, and MUFA-enriched membranes resist lipid peroxidation [20]. On this reading, the determinant is not CYB5R3 abundance but which acceptor pathway is available to consume its electrons.

This remains a hypothesis. The evidence assembled here is compatible with it and does not demonstrate it. Differential electron routing, ferroptosis resistance, and ER-stress activation were not measured; what was measured is expression, survival, and a set of weak pairwise associations whose tissue difference required formal testing (Section 2.6). Where that test does not survive correction, the associations are consistent with the hypothesis rather than evidence for it. The falsifiable predictions that would decide the question are set out in Section 3.6.

### 3.3. The Human Data Do Not Support Reinforcing CYB5R3 in MASLD

The case for reinforcing CYB5R3 early rests on causal gain-of-function experiments in mice, in which transgenic over-expression accumulates long-chain polyunsaturated fatty acids, improves mitochondrial function, reduces oxidative damage and inflammation, and reduces diethylnitrosamine-induced carcinogenesis, and in which co-expression with NQO1 reproduces features of caloric restriction [6,7]. Those results stand.

What the present analysis addresses is the corollary usually assumed alongside them, that endogenous hepatic CYB5R3 becomes deficient as steatotic liver disease advances. Across the three human liver-biopsy cohorts examined here, the relationship between *CYB5R3* expression and histological severity does not replicate: the cohorts disagree on its sign, and the pooled estimate against fibrosis stage is +0.01 with I^2^ = 83%. The corollary is therefore neither confirmed nor refuted; it is unsupported. The heterogeneity is itself informative, because it is specific. *MTARC1*, *PARP16*, and *SCD*, measured in the same biopsies against fibrosis stage, each give a consistent pooled estimate with I^2^ = 0%, whereas against the NAFLD activity score all four—these three and *CYB5R3*—are heterogeneous. This is consistent with the inconsistency in *CYB5R3* being a property of that transcript in liver tissue rather than of the cohorts, though it does not establish it: the fibrogenic positive controls were themselves heterogeneous against fibrosis stage (I^2^ = 62% and 53%), so cohort differences are real, I^2^ is estimated from only three cohorts and is correspondingly imprecise, and no test of the difference in heterogeneity between transcripts was performed. I do not have data that identify the cause. Cell-composition shifts, the narrow dynamic range of a highly expressed housekeeping-like transcript, and post-transcriptional regulation of the kind documented for CYB5R3 [16] are all candidates. Supraphysiological overexpression in a mouse and the endogenous gradient in a human biopsy are therefore measuring different things, and only the former speaks to therapeutic benefit.

Two practical consequences follow. *CYB5R3* transcript level should not be used as a biomarker of lost protection in MASLD. And any proposal to raise CYB5R3 activity in patients would have to be tested directly against histological endpoints rather than inferred from expression data, while reckoning with the fact that the same electron output supplies SCD1. I note the concern, raised in review, that indiscriminate enhancement of CYB5R3 could fuel lipogenesis and, if mistimed, tumor development. The human data reported here are consistent with that concern, and the therapeutic proposals of the original submission have been withdrawn accordingly. Section 3.6 now contains falsifiable predictions and no intervention scheme.

### 3.4. Relationship to the mARC1 Literature, Including Its Contradictions

The original submission proposed inhibiting mARC1 as a therapeutic tactic. That proposal is withdrawn, for two reasons that were correctly identified in review.

It contradicted the present data. Higher *MTARC1* expression was associated with longer survival in the external cohort before covariate adjustment, so proposing to lower it in established HCC is not consistent with the analysis. The physical coupling of the two enzymes tells against the opposite proposal as well: if CYB5R3 and mARC1 share one electron relay, then raising CYB5R3 early should raise mARC1 activity, which is the direction that human genetics identifies as harmful [21,22,23]. Neither arm of the original program survives its own premise.

The proposal was also not new. Three in vivo programs have already tested hepatic mARC1 loss. Hepatocyte-targeted GalNAc-siRNA knockdown reduced steatosis and hepatic lipid content but left Sirius Red fibrosis and collagen 1 unchanged, although α-smooth-muscle actin staining and several fibrogenic transcripts did fall [24]. Liver-specific *Mtarc1* knockdown reduced transaminases, hepatic triglycerides, and inflammation across two diet-induced MASH models, with only a minimal effect on collagen area [25]. Global *Mtarc1* knockout reduced fibrosis by close to 50% in a 32-week GAN-diet model and by 24% in a 20-week high-fat, high-fructose model, and, critically, GalNAc-siRNA given after 16 weeks of GAN diet reduced collagen, whereas the same treatment given after 24 weeks, at higher disease burden, had no effect on any liver endpoint; in the same study, a hepatocyte-specific knockdown had no effect on fibrosis in a choline-deficient high-fat model, although global knockout did reduce fibrosis in that model [26]. Two further 2026 studies report protection from steatotic liver disease in *Mtarc1*-deficient mice [27,28], the second attributing it to remodeling of lipid-droplet phospholipids [28].

The literature is not unanimous, and the manuscript now says so. Whole-body *Mtarc1* knockout mice were not protected from hepatic triglyceride accumulation, inflammation, or fibrosis in one large study, whose authors attribute the discrepancy to the paralog *Mtarc2* being the principal mARC enzyme in mouse liver [29]. A knock-in mouse carrying the murine equivalent of the protective human A165T substitution reduced mARC1 protein by approximately 50–80% without changing mRNA, yet showed no reduction in histological steatosis, inflammation, or fibrosis in any of three diet models, in both sexes in the two models that used both, although liver triglyceride content did fall in one of them, which implies that partial protein reduction is insufficient [30]. Any account of this axis has to accommodate both sets of results, and citing only the protective side would misrepresent the field.

### 3.5. Limitations

This study is observational and in silico. Expression–prognosis associations do not establish causality, and no functional experiment was performed.

Neither TCGA-LIHC as harmonized in UCSC Xena nor GSE14520 supplies systemic-therapy data, so no model here is adjusted for treatment, and treatment differences between expression strata cannot be excluded. Cohort sizes were fixed by the public resources, and no sample-size or power calculation was performed; the study is therefore powered by whatever was available rather than by design. The effect sizes are modest, and the clinical increment is not demonstrated. The adjusted hazard ratio of 1.23 per standard deviation corresponds to a small shift in risk; the ΔC-index over a model containing age, sex, and stage is 0.005 with a confidence interval spanning zero, and the association loses significance when vascular invasion or cirrhosis is added, albeit in smaller subsets. *CYB5R3* should not be described as a prognostic biomarker on this evidence.

The correlations underpinning the tissue-context hypothesis are weak, account for a small fraction of between-patient variance, and are subject to confounding by tumor cellular composition; the formal tests, the adjustment for tumor cellular composition (a stromal and immune marker proxy, not an ABSOLUTE purity call), and the permutation null in Section 2.6 are the relevant evidence, and where they do not survive correction, the hypothesis is unsupported by that particular line of evidence.

Two findings that argue against a simple tumor-supportive role are carried here rather than mentioned once. *CYB5R3* expression shows a decreasing trend across histologic grade in TCGA-LIHC, which is difficult to reconcile with high expression marking worse survival, and the direction of that tension is not resolved by anything reported here. And CYB5R3 is not a genetic dependency in liver cancer cell lines, so any tumor-supportive role would have to be conditional on the surrounding metabolic context rather than intrinsic.

The liver-versus-lung contrast rests on a single lung cohort. I was not able to identify a lung adenocarcinoma series with matched expression and survival that would support an equivalently specified replication of the correlation analysis, so the tissue difference reported in Section 2.6 is internally consistent but externally unreplicated.

No metabolically defined HCC population is analyzed anywhere in this study. The TCGA-LIHC clinical annotation records positive viral serologies but does not record their absence, so a non-viral or metabolic subset cannot be constructed from it (Section 2.9), and no public HCC cohort with both MASH etiology and survival was available to me. Since the prognostic claim concerns HCC in general while the motivating question concerns metabolic liver disease, this gap is the single most important limitation of the study.

Resource independence is limited. UALCAN, cBioPortal, KM Plotter’s liver cohort, and the pan-cancer scan share TCGA patients. GSE14520 is the only independent survival cohort; it is HBV-related and predominantly Chinese, and generalization to metabolic HCC is not established.

The MASLD analysis rests on bulk tissue, so cellular composition changes with disease severity and can itself drive transcript associations; deconvolution of one of these cohorts (GSE162694) has shown hepatocyte content falling and stellate-cell, macrophage, and cholangiocyte content rising as fibrosis advances [19]. The pathologist-assigned grades are the more reliable readout, but they were recorded using different scoring systems across cohorts. For *CYB5R3*, the three cohort estimates against fibrosis stage differ in sign (+0.25, +0.01, −0.22; I^2^ = 83%), and no claim about its relationship to severity is made in either direction. I report this rather than selecting the cohort that supports the original hypothesis, which a single-cohort analysis would have done. The fibrogenic controls were also somewhat heterogeneous (*COL1A1* I^2^ = 62%, *TGFB1* I^2^ = 53%), so cohort differences are real; but they cannot explain the *CYB5R3* result on their own, since *MTARC1*, *PARP16*, and *SCD* give I^2^ = 0% in the same comparisons.

The CPTAC protein estimate is lower in tumor, while two of the four transcript estimates are higher, so whether transcript and protein diverge for CYB5R3 in HCC depends on which transcript pipeline is used, and the question is not settled here. If a divergence exists, post-translational regulation, including the ufmylation pathway [16], is one candidate; differences in cohort, platform, tumor purity and cellular composition between the transcriptomic and proteomic series are equally plausible.

Finally, on tractability. CYB5R3 belongs to a family of closely related reductases, of which CYB5R1, CYB5R2, and CYB5R4 remain comparatively understudied [4], so isoform selectivity for any pharmacological agent is an open question rather than a solved problem, and I am not aware of published work addressing it. On-target risk is also real: biallelic *CYB5R3* deficiency causes recessive congenital methemoglobinemia, in which the generalized type II form produces severe and irreversible neurological disease [31]. Neither consideration was addressed in the original submission, and both bear directly on the tractability of CYB5R3 as a drug target in either direction: the HCC association reported here would point toward inhibition, whereas the mouse gain-of-function literature has been read as an argument for reinforcement.

### 3.6. Future Perspectives

The model yields predictions that would falsify it. In matched HCC and lung cell lines, modulating CYB5R3 should change ER-stress readouts (PARP16, phosphorylated PERK and IRE1α, GSSG) and lipogenic and ferroptosis readouts (SCD1, MUFA-to-SFA ratio, sensitivity to RSL3 or erastin with and without ferrostatin) in opposite directions, alongside NAD^+^/NADH. *PARP16* knockdown should abolish the tumor-suppressive effect of CYB5R3 in the lung. SCD1 inhibition should convert *CYB5R3*-high HCC cells from ferroptosis-resistant to ferroptosis-sensitive. In human liver, single-cell or spatial data should show whether the divergence of *CYB5R3* and *MTARC1* across histological lesions reflects hepatocyte biology or shifting cellular composition, which bulk tissue cannot distinguish. A null result in any of these would count against the model, and I regard that as the point of stating them.

## 4. Materials and Methods

### 4.1. Design and Reporting

This study used only publicly available, de-identified datasets; no new human or animal data were generated, so ethical approval was not required. The study is exploratory and hypothesis-generating. Reporting follows REMARK for the prognostic-biomarker analyses and STROBE for the observational analyses; completed checklists are provided as Supplementary Files S1 and S2, with the corresponding references [32,33].

### 4.2. Data Sources and Versions

Raw matrices were obtained from UCSC Xena [34]: TCGA.LIHC.sampleMap/HiSeqV2 and TCGA.LUAD.sampleMap/HiSeqV2 (log_2_(norm_count + 1)); TCGA.LIHC.sampleMap/LIHC_clinicalMatrix; the TOIL recompute [35] TcgaTargetGtex_rsem_gene_tpm with TcgaTargetGTEX_phenotype.txt (log_2_(TPM + 0.001)), which combines TCGA [36] and GTEx [37] samples on one pipeline; and, from the PanCanAtlas hub, EB++AdjustPANCAN_IlluminaHiSeq_RNASeqV2.geneExp.xena, the TCGA Clinical Data Resource survival table [38]. An ABSOLUTE consensus tumor-purity table (TCGA_mastercalls.abs_tables_JSedit.fixed.txt) was also queried from that hub but could not be resolved under the identifier used on the deposited run, and was therefore not used (Section 4.6). GEO series GSE14520, GSE130970, GSE135251, and GSE162694 were retrieved with GEOquery version 2.80.0. [39]. DepMap Chronos gene-effect scores [40] were obtained through the Bioconductor depmap package, version 1.26.0, with ExperimentHub 3.2.0. The ExperimentHub release is resolved at run time rather than pinned in the script; the deposited run resolved to the snapshot of 21 April 2026, which the package reports at load time and which is therefore recorded in the deposited run log rather than in a result file. Portal values were retrieved from UALCAN, KM Plotter, cBioPortal, and the Human Protein Atlas on 15 July 2026, and all raw-data analyses were run on the dates recorded in the run logs of the deposited pipeline; the previously inconsistent access dates have been reconciled there. The official symbol *MTARC1* is indexed as *MARC1* or *MOSC1* in several legacy resources; the aliases queried are fixed in the shared setup file, and the alias that succeeded is recorded in the alias_used column of results/10_expression_estimates.csv for the tumor-versus-normal matrices. For the TOIL retrieval used in the survival analyses, it is recorded in the note column of results/11_flow.csv; for the remaining retrievals, the alias and any lookup failure appear in the deposited run log rather than in a result file.

### 4.3. Nomenclature

Gene symbols are italicized and follow HGNC: *CYB5R3*, *MTARC1*, *SCD*. Protein names are set in roman: CYB5R3, mARC1, SCD1. Where the literature uses *SCD1* for the gene, the HGNC symbol *SCD* is used here, and the equivalence is noted at first mention.

### 4.4. Expression Analyses

Sample selection differs by matrix, and the difference accounts for most of the differing counts in Table 2; the remainder is a difference in matrix membership, noted below. On the two HiSeqV2 matrices, tumor samples were identified by TCGA sample-type codes 01, 02, 03, 05, 06, and 07 and adjacent normal by code 11; in these two cohorts the qualifying samples are overwhelmingly code 01 (primary solid tumor), and the text refers to these sets as primary tumors on that basis. On the TOIL recompute and the PanCanAtlas matrix, which are indexed by phenotype rather than by barcode, samples were selected by case-insensitive matching of the phenotype field against Primary Tumor for tumors and against Normal for the adjacent-normal reference, within TCGA samples only, which is a slightly stricter rule than the barcode codes; this is why the TOIL tumor count is 369 where the HiSeqV2 count is 373, and 513 where the LUAD HiSeqV2 count is 517. The PanCanAtlas matrix applies the same phenotype rule but is a separately assembled release, and one LUAD tumor present on the TOIL matrix is absent from it, which is why its LUAD count is 512 rather than 513; the LIHC counts on the two matrices agree. Where an analysis is at the patient level, one sample per patient was retained by taking the first sample for that patient in the order in which the matrix presents its columns; two LIHC patients and two LUAD patients contribute a second tumor sample and are affected by this rule. The expression comparison uses every qualifying sample, so its counts are samples; the analyses that are at the patient level additionally retain one sample per patient. This is why the survival analyses in Section 2.3 begin with 371 patients rather than the 373 LIHC samples in the HiSeqV2 matrix, and why the correlation analyses of Section 2.6 use 515 LUAD patients rather than the 517 LUAD samples of Table 2. Group comparisons used the Wilcoxon rank-sum test with the median difference on the log_2_ scale as the effect estimate and Cliff’s delta as a non-parametric effect size. Because matrices with different normalizations and different normal references give different answers to the same question, all four estimates are reported together (Table 2), the reference estimate is designated in advance, and no value is transcribed from a rendered plot.

### 4.5. Survival Analyses

*CYB5R3* expression for the survival analyses was taken from the TCGA-LIHC HiSeqV2 matrix and *MTARC1* from the Xena TOIL matrix, because *MTARC1* could not be retrieved from the legacy HiSeqV2 probe map under any of its aliases (Section 2.2). Each gene was standardized to unit standard deviation within the analysis set of each model rather than once across the whole cohort, so “per standard deviation” refers to the standard deviation of the set actually modeled; the extended-covariate estimates in Section 2.3 are therefore not on exactly the same scale as the primary estimate. The single-gene models are therefore internally consistent, but the joint model, the likelihood-ratio test, the interaction term, the difference of standardized expressions, and the between-gene correlation combine two differently normalized pipelines; all of these are labeled exploratory for that reason, and the limitation is stated in the Conclusions. The primary analysis was a Cox proportional-hazards model of overall survival on expression standardized to unit standard deviation, adjusted for age, sex, and AJCC stage dichotomized as I–II versus III–IV. That covariate set was fixed before the models were fitted: age, sex, and stage are the demographic and staging variables recorded for almost the whole cohort and are the established determinants of survival in hepatocellular carcinoma. No covariate was chosen on the basis of its association with the outcome in these data, and none was added or removed after seeing a result. Patient flow, number of events, median follow-up by reverse Kaplan–Meier, and the comparison of included with excluded patients are reported in Section 2.3. Every term in the primary models, including age, sex, and stage, is deposited with its hazard ratio, 95% confidence interval, and *p*-value in results/11_cox_primary_full_coefficients.csv, as the REMARK checklist requires. Proportional hazards were assessed with scaled Schoenfeld residuals for each covariate and globally. A non-linear expression effect was tested by comparing the linear term with a penalized spline of three degrees of freedom; the reference degrees of freedom for the likelihood-ratio test are the fractional effective degrees of freedom of the fitted spline, which is why the test is reported on 2.05 rather than 2 degrees of freedom. Discrimination was quantified by Harrell’s C-index for a clinical base model and for the base model plus expression, with the difference and its bootstrap percentile confidence interval from 1000 resamples. Both models are refitted and evaluated on each bootstrap sample, so the reported C-indices are apparent values without an optimism correction; since the conclusion drawn from them is that the increment is negligible, an optimism correction would move the estimate in the conservative direction. Missing covariates were handled by complete-case analysis for the primary model, with multiple imputation by chained equations (m = 20, including the Nelson–Aalen cumulative hazard as an auxiliary variable) pre-specified as a sensitivity analysis. The two-axis members were modeled jointly, with a likelihood-ratio test against the single-gene model and a multiplicative interaction term. A median split, with ties assigned to the high group, is reported as a secondary analysis and labeled as such. The optimal-cut-off estimates that appear in Section 2.3 and Table 3 are portal outputs from KM Plotter, not pipeline analyses. Progression-free interval, taken from the TCGA Clinical Data Resource, was analyzed as a sensitivity endpoint with the same covariates on its own complete-case set. For GSE14520, probe sets annotated to each gene symbol were averaged, the series matrices were used as deposited, and the endpoint was overall survival in months. Unadjusted, stage-adjusted, and extended models were specified in advance; serum α-fetoprotein was among the covariates specified for the extended model but is recorded in that series as a single category and was therefore dropped automatically, so the extended model contains age, sex, TNM stage, cirrhosis, and multinodularity only. The series comprises two Affymetrix platforms, each normalized within itself by the depositors; the matrices were combined without cross-platform normalization, and no platform term was included, which is recorded as a limitation in the Conclusions.

### 4.6. Correlation Analyses

Spearman correlations were computed within primary tumors of each cohort, with Fisher-z 95% confidence intervals and Benjamini–Hochberg q-values across the pre-specified panel. The difference between the liver and lung coefficients was tested by Fisher’s r-to-z transformation with Benjamini–Hochberg correction across the panel. Fisher-z variances were computed as 1/(*n* − 3), the Pearson expression, without a Spearman-specific variance correction; the intervals are therefore marginally narrower, and the z statistics marginally larger, than a Spearman-specific variance would give. Partial Spearman correlations were computed on rank-transformed variables, conditioning on tumor cellular composition. The pipeline uses the ABSOLUTE consensus purity call only when at least 100 tumors of a cohort carry one, and falls back to a marker-based proxy otherwise; on the deposited run, the ABSOLUTE table could not be resolved on the Xena hubs under the identifier queried, so no cohort met that threshold and the fallback was used in both. The number of matched calls appears in the deposited run log rather than in a result file. The conditioning variable was therefore the first principal component of a panel of established stromal and immune marker genes, with each marker standardized to zero mean and unit variance and any missing value set to that marker’s own mean, and the component sign flipped so that higher values are intended to indicate higher tumor content. Partial correlations are invariant to that sign, so the orientation affects interpretation of the covariate only, not any reported coefficient. This is a weaker adjustment than a purity call, and it is described as such wherever it is used; the manuscript does not claim to have adjusted for tumor purity. The composition-adjusted coefficients are corrected within their own Benjamini–Hochberg families: one of 16 tests within each cohort and one of 16 tests for the adjusted tissue-difference comparison; the adjusted q-values quoted in Section 2.6 come from those families and not from the unadjusted ones. A permutation null for the number of sign reversals was generated by permuting *CYB5R3* expression within each cohort 10,000 times. Terms such as “module” are avoided; the results are exploratory pairwise associations.

### 4.7. Human MASLD Liver-Biopsy Cohorts

Hepatic expression of *CYB5R3*, *MTARC1*, *SCD*, *NQO1*, *PARP16*, *FASN*, *COL1A1*, and *TGFB1* was examined in GSE130970 (78 biopsies with grades for steatosis 0–3, lobular inflammation 0–2, cytological ballooning 0–2, NAFLD activity score 0–7 and fibrosis stage 0–4) [17], GSE135251 (216 biopsies with NAFLD activity score and fibrosis stage) [18] and GSE162694 (143 biopsies, of which 128 could be matched to their sample annotation; among the matched samples 99 carried a fibrosis stage and 102 a NAFLD activity score in the deposited annotation, and only samples with a recorded grade enter the corresponding test) [19]. Sample identifiers in GSE162694 do not correspond to GEO accessions, so the pipeline scores every candidate annotation column by normalized exact match and, failing that, by bidirectional substring containment, adopts the best-scoring column as the key and requires the match to be unique; on the deposited run, the sample title was the column selected, at 89.5% coverage. Which column was chosen appears in the deposited run log rather than in a result file; the pipeline records the number of samples matched, from which the 15 unmatched follow, and the matched samples themselves are deposited in results/14_masld_sample_level.csv. Counts were converted to counts per million where the deposited matrix was not already normalized, and values were log_2_(x + 1)-transformed. Where a gene symbol maps to more than one row of a deposited matrix, the first matching row was used rather than an average; this differs from the probe averaging used for GSE14520 and is recorded here because the two conventions are not the same. Associations with ordinal grades were tested by Spearman rank correlation, with Benjamini–Hochberg correction across all 72 gene × histology × cohort tests. Kruskal–Wallis statistics were computed alongside them and are reported uncorrected in the deposited output; they are not part of the corrected family, and no claim rests on them. To separate the histological components, a linear model containing steatosis, lobular inflammation and ballooning, each entered as a numeric score, was fitted in GSE130970, the only cohort that grades the three lesions separately, together with partial correlations adjusting for the remaining two lesions. The coefficients of that model and the partial correlations are corrected together in a single Benjamini–Hochberg family of 48 tests, separate from the 72-test cohort-level family; the q-values quoted in Section 2.8 for these terms come from it. Cohort estimates were pooled by random-effects meta-analysis (restricted maximum likelihood) of Fisher-z-transformed coefficients, with I^2^ reported. The pooled estimates form a third, separate Benjamini–Hochberg family of 16 tests (eight genes × two pooled endpoints); the pooled q-values in Table 5 are corrected within that family and not within the 72-test cohort-level family. Because the number of cohorts is small, the between-study variance is estimated imprecisely, and the cohort-specific estimates are given alongside every pooled value so that heterogeneity is visible directly rather than only summarized.

### 4.8. Pan-Cancer and Dependency Analyses

Cohorts with at least 20 evaluable tumors and 10 deaths were analyzed by univariable Cox regression of overall survival on standardized expression; tumor-versus-normal comparisons required at least 10 adjacent-normal samples. Twenty-eight cohorts met the survival criteria, and 15 of them also had enough adjacent-normal samples for the expression comparison. Benjamini–Hochberg correction was applied within each family separately, across 28 cohorts for survival and 15 for expression. The LIHC row is flagged in the output as drawing on the same TCGA-LIHC patient population as the primary analysis, and therefore as not being an independent replication of it. DepMap lineages, defined by the DepMap lineage annotation rather than by TCGA histology, were compared by a two-sided Wilcoxon rank-sum test, with the percentage of lines below the −0.5 Chronos threshold recorded in results/15_depmap_dependency.csv; the counts quoted in Section 2.5 follow from that percentage and the number of lines in each lineage. Stage and histologic grade were tested for a monotone trend by the two-sided Jonckheere–Terpstra test. Within each etiology group, the prognostic value of *CYB5R3* was assessed by univariable Cox regression on standardized expression, without covariate adjustment, and a group was analyzed only if it contained at least 10 patients and 3 deaths. Etiology was classified from the clinical columns whose names match viral hepatitis serology, HBV, or HCV, of which viral_hepatitis_serology is the one populated in this release; these fields list serologies returning a positive result. Their values were concatenated per patient, placeholders for missing or unknown values were discarded, and a patient with any remaining value was taken as viral serology positive and one with none as no positive viral serology recorded, a category that cannot be distinguished from untested and is therefore not treated as non-viral or metabolic.

### 4.9. Software and Reproducibility

All analyses were performed in R version 4.6.1 (R Foundation for Statistical Computing, Vienna, Austria) with UCSCXenaTools 1.7.0 [41], GEOquery 2.80.0 [39], survival 3.8-6 [42], mice 3.19.0 [43], metafor 5.0-1 [44], clinfun 1.1.5, data.table 1.18.4, dplyr 1.2.1, and ggplot2 4.0.3; the version of every package is also recorded in the deposited sessionInfo files. Cox models, proportional-hazards diagnostics, the penalized spline, and the concordance index were computed with survival [42]; multiple imputation with mice [43]; random-effects meta-analysis with metafor [44]; and the Jonckheere–Terpstra trend tests with clinfun. Every figure produced by the pipeline is drawn at 300 dpi, at the width given in its save_fig call—180 mm for most, 170 mm for Supplementary Figure S2, and 150 mm for Supplementary Figures S3, S5 and S6—and the deposited helper tools/retag_tiff.py then stamps that resolution into the PNG and writes an LZW-compressed TIFF of the same pixels beside it, because the R raster devices on the machine used here omit the resolution metadata from both formats. Figure 1 and Figure 6 are schematics that the pipeline does not draw; they are supplied at the same resolution. Unless stated otherwise, tests were run at their default settings: Wilcoxon rank-sum tests use the normal approximation with continuity correction, Spearman tests use the asymptotic t approximation rather than the exact permutation distribution, which the pipeline requests explicitly, the one exception being the 15-cohort correlation of fold change against hazard ratio in Supplementary Figure S3, which is left at the function default and, being free of ties, is therefore computed exactly, Cox models use the Efron approximation for ties, and proportional-hazards diagnostics use the Kaplan–Meier time transform. A single random seed is fixed when the shared setup file is loaded, and the permutation null and the multiple imputation each re-seed to the same value immediately before running, so both reproduce independently of execution order. The bootstrap that produces the C-index interval does not re-seed and draws from the state the preceding analyses leave, so that interval reproduces exactly only when the pipeline is run in its scripted order. The pipeline uses mice, metafor, and clinfun, but does not stop when one is absent: without metafor it falls back to a fixed-effect inverse-variance pooling, without clinfun to a Kruskal–Wallis test, and without mice it skips the multiple-imputation sensitivity analysis altogether and writes no result for it. A reproducer must therefore confirm from the session information that all three were installed before comparing output with what is reported here. The complete pipeline, including scripts, sample-selection criteria, preprocessing steps, model formulas, the numerical source data underlying every figure and table entry computed in this study, run logs, and session information, is deposited in a public repository and archived with a citable DOI (Data Availability).

## 5. Conclusions

In TCGA-LIHC, higher *CYB5R3* expression was associated with shorter overall survival independently of age, sex, and stage, and the direction was reproduced in the one independent cohort examined, in which higher *MTARC1* was associated with longer survival before, but not after, covariate adjustment. In three human liver-biopsy cohorts spanning the MASLD spectrum, *MTARC1* fell with fibrosis stage reproducibly and without detectable heterogeneity, whereas *CYB5R3* gave estimates that differed in sign between the same cohorts, so no relationship between *CYB5R3* expression and histological severity is established. The two members of a physically coupled electron-transfer relay therefore behave differently, before and after malignant transformation, and no correlation between them was detectable within tumors. That difference in behavior is the finding of this study. It rests on each gene’s own estimate rather than on a formal test of the contrast between them, which was not performed.

The most important limitation is that no metabolically defined HCC population is analyzed anywhere in this study, so the question that motivated the work is not answered by it. A further limitation is that every claim here is associative, and the prognostic association is small. *CYB5R3* adds essentially nothing to age, sex, and stage (ΔC-index 0.005, 95% CI −0.015 to +0.037), the external single-gene association does not survive covariate adjustment, no TCGA cohort survives correction for multiplicity, it is not a genetic dependency in liver cancer cell lines, its protein is not more abundant in tumor than in normal liver, and its expression shows a decreasing trend across histologic grade. Two technical limitations bear on specific analyses. *CYB5R3* and *MTARC1* are measured on different Xena matrices, because *MTARC1* is absent from the legacy HiSeqV2 probe map, so every term that combines the two genes—the joint model, the interaction, the difference of standardized expressions, and the between-gene correlation—combines two differently normalized pipelines and is reported as exploratory on that ground. GSE14520, the only independent survival cohort, comprises two Affymetrix platforms that were combined as deposited without cross-platform normalization or a platform term, so a platform effect cannot be excluded from the external estimates. A tissue-context model, in which the fate of CYB5R3’s electron output is set by the acceptor pathways available locally, remains a hypothesis that this analysis motivates but does not establish. The therapeutic proposals of the original submission have been withdrawn because the human data do not support them.

## Figures and Tables

**Table 1:** Separation of portal-derived observations from analyses performed for this study.

Section	Question	Source	Status
Section 2.2	Tumor-versus-normal expression, LIHC and LUAD	UCSC Xena raw matrices (TCGA HiSeqV2; TOIL TCGA+GTEx; PanCanAtlas EB++)	Analyzed here from raw data
Section 2.2	Same question, portal rendering	UALCAN	Portal output, reported for comparison only
Section 2.3	Independent prognostic value	UCSC Xena + TCGA-CDR	Analyzed here (primary analysis)
Section 2.3	Portal survival splits	UALCAN (upper quartile), KM Plotter (optimal cut-off)	Portal output, secondary
Section 2.4	External replication	GEO GSE14520	Analyzed here; only independent survival cohort
Section 2.5	Protein, copy number	CPTAC via UALCAN; cBioPortal	Portal output
Section 2.5	CRISPR dependency	DepMap (Chronos)	Analyzed here; negative result
Section 2.6	Pairwise associations, liver versus lung	UCSC Xena raw matrices	Analyzed here, with formal tissue test
Section 2.7	Pan-cancer scan	UCSC Xena PanCanAtlas + TCGA-CDR	Analyzed here, FDR-corrected across cohorts
Section 2.8	MASLD histological spectrum	GEO GSE130970, GSE135251, GSE162694	Analyzed here
Section 2.9	Stage, grade, etiology	UCSC Xena TCGA-LIHC	Analyzed here

**Table 2:** Reconciliation of the four raw-data tumor-versus-normal estimates for *CYB5R3*, with the portal rendering shown for comparison. log_2_FC is the difference of the tumor and normal medians on the log_2_ scale of each matrix, and the medians themselves are deposited in results/10_expression_estimates.csv; δ is Cliff’s delta. Counts here are tumor samples; the survival analyses in Section 2.3 retain one sample per patient and therefore begin from 371 patients rather than 373 samples on the HiSeqV2 matrix (Section 4.4). Full numbers, including interquartile ranges and the *MTARC1* estimates, are in results/10_expression_estimates.csv.

Cohort	Matrix and Normalization	Normal Reference	*n* Tumor/Normal	log_2_FC	Cliff’s δ	Wilcoxon *p*	Role
LIHC	Xena TOIL RSEM, log_2_(TPM + 0.001)	GTEx normal liver	369/110	+0.213	+0.198	0.0016	Reference (pre-designated)
LIHC	Xena TOIL RSEM, log_2_(TPM + 0.001)	TCGA adjacent normal	369/50	+0.481	+0.432	7.1 × 10^−7^	Sensitivity
LIHC	TCGA HiSeqV2, log_2_(norm_count + 1)	TCGA adjacent normal	373/50	−0.070	−0.148	0.088	Sensitivity
LIHC	PanCanAtlas EB++ batch-corrected	TCGA adjacent normal	369/50	−0.060	−0.143	0.102	Sensitivity (Supplementary Table S1)
LIHC	UALCAN, TCGA level-3 TPM, *t*-test	TCGA adjacent normal	371/50	not exported	—	<1 × 10^−12^	Portal output only
LUAD	Xena TOIL RSEM, log_2_(TPM + 0.001)	GTEx normal lung	513/288	−0.731	−0.716	1.2 × 10^−63^	Reference
LUAD	Xena TOIL RSEM, log_2_(TPM + 0.001)	TCGA adjacent normal	513/59	−0.929	−0.808	2.6 × 10^−24^	Sensitivity
LUAD	TCGA HiSeqV2, log_2_(norm_count + 1)	TCGA adjacent normal	517/59	−0.820	−0.816	9.4 × 10^−25^	Sensitivity
LUAD	PanCanAtlas EB++ batch-corrected	TCGA adjacent normal	512/59	−0.815	−0.814	1.3 × 10^−24^	Sensitivity

**Table 3:** Portal-derived observations, reported for comparison only. Three of these four resources (UALCAN, KM Plotter, and cBioPortal) render TCGA data, so agreement among them measures agreement between renderings of one dataset (Section 2.1); the CPTAC proteomic contrast derives from an independent HBV-related cohort. Hazard ratios are shown with their confidence intervals and *p*-values wherever they appear in this manuscript.

Portal	Analysis	Result	Statistical Significance
UALCAN	*CYB5R3*, LIHC tumor versus adjacent normal	higher in tumor	*p* < 1 × 10^−12^
UALCAN	*MTARC1*, LIHC tumor versus adjacent normal	lower in tumor	*p* = 0.015
UALCAN	*CYB5R3*, LIHC overall survival, upper-quartile split (high *n* = 91 versus low/medium *n* = 274)	worse with high expression	*p* = 0.001
UALCAN	*CYB5R3*, LUAD overall survival	no stratification	*p* = 0.87
KM Plotter	*CYB5R3*, LIHC overall survival, optimal cut-off (*n* = 364)	HR 1.24 (95% CI 0.86–1.81)	log-rank *p* = 0.25, not significant
KM Plotter	*MTARC1*, LIHC overall survival, optimal cut-off (*n* = 364)	HR 0.57 (95% CI 0.40–0.80)	log-rank *p* = 0.0013
CPTAC via UALCAN	CYB5R3 protein, HCC versus adjacent normal (*n* = 165/165)	lower in tumor (median Z ≈ 0.0 versus ≈ +0.35)	*p* = 1.3 × 10^−5^
cBioPortal	*CYB5R3* mRNA against copy-number status	monotone increase (shallow deletion < diploid < gain)	—
cBioPortal	Somatic *CYB5R3* or *SCD* alteration frequency	approximately 1% of cases	—

**Table 4:** Exploratory pairwise associations of *CYB5R3* with the pre-specified 16-gene redox panel in TCGA-LIHC (*n* = 371) and TCGA-LUAD (*n* = 515), with the formal test of the tissue difference. r_s_, Spearman coefficient; q, Benjamini–Hochberg-corrected p within each cohort; q(diff), BH-corrected p for the Fisher r-to-z comparison across the panel. Rows are ordered by q(diff). Confidence intervals for every coefficient, and the composition-adjusted values, are in results/12_coexpression_full.csv and results/12_purity_adjusted.csv.

Gene	Functional Class	LIHC r_s_	LIHC q	LUAD r_s_	LUAD q	z (Diff)	q (Diff)	Verdict
*CAT*	ROS scavenger	−0.105	0.100	+0.225	4 × 10^−6^	−4.88	1.7 × 10^−5^	significant reversal
*PARP16*	Pro-death ER stress	−0.131	0.086	+0.115	0.028	−3.62	0.0024	significant reversal
*TXNRD1*	Thioredoxin	+0.049	0.439	−0.169	0.001	+3.22	0.0069	significant reversal
*SOD2*	ROS scavenger	+0.108	0.100	−0.106	0.044	+3.13	0.0070	significant reversal
*SCD*	Lipogenic desaturase	+0.164	0.024	−0.041	0.516	+3.02	0.0081	significant reversal
*KEAP1*	NRF2 axis	−0.093	0.133	+0.088	0.090	−2.66	0.021	significant reversal
*GPX4*	Ferroptosis defense	−0.029	0.667	+0.089	0.090	−1.72	0.195	apparent reversal, not significant
*PRDX1*	Peroxiredoxin	−0.119	0.086	−0.011	0.859	−1.59	0.222	no significant difference
*NQO1*	NRF2 target	+0.114	0.090	+0.019	0.788	+1.40	0.285	no significant difference
*GCLM*	Glutathione	−0.069	0.269	−0.149	0.003	+1.19	0.342	no significant difference
*GSR*	Glutathione	+0.089	0.141	+0.007	0.875	+1.20	0.342	no significant difference
*G6PD*	NADPH/PPP	+0.014	0.833	+0.069	0.211	−0.80	0.487	no significant difference
*GCLC*	Glutathione	−0.122	0.086	−0.062	0.254	−0.89	0.487	no significant difference
*TXN*	Thioredoxin	−0.095	0.133	−0.150	0.003	+0.82	0.487	no significant difference
*SOD1*	ROS scavenger	−0.048	0.439	−0.018	0.788	−0.44	0.701	no significant difference
*NFE2L2*	NRF2 axis	−0.001	0.978	+0.019	0.788	−0.30	0.763	apparent reversal, not significant

**Table 5:** *CYB5R3*, *MTARC1* and adjacent genes against pathologist-assigned histology in three human liver-biopsy cohorts. r_s_, Spearman coefficient; the per-cohort q is Benjamini–Hochberg-corrected across the 72 gene × histology × cohort tests, and the pooled q across a separate family of the 16 pooled meta-analytic tests (Section 4.7). Pooled estimates are random-effects meta-analyses of Fisher-z-transformed coefficients with I^2^ for between-cohort heterogeneity. Only features graded in more than one cohort are pooled; the ballooning, steatosis and lobular-inflammation rows are graded in GSE130970 alone and are therefore not pooled. Both histological endpoints are shown for every gene except the two fibrogenic positive controls, whose full results are in the deposited output. Full results are in results/14_masld_trend_all.csv and results/14_masld_meta.csv.

Gene	Histological Feature	GSE130970 (*n* = 78)	GSE135251 (*n* = 216)	GSE162694 (*n* = 99–102)	Pooled r_s_ (95% CI)	Pooled q	I^2^
*CYB5R3*	fibrosis stage	+0.246 (0.068)	+0.010 (0.93)	−0.221 (0.065)	+0.008 (−0.243 to +0.258)	0.95	83%
*CYB5R3*	NAFLD activity score	+0.333 (0.012)	+0.127 (0.12)	−0.131 (0.29)	+0.110 (−0.147 to +0.353)	0.50	83%
*CYB5R3*	ballooning grade	+0.313 (0.019)	not graded	not graded	—	—	—
*CYB5R3*	steatosis grade	+0.263 (0.048)	not graded	not graded	—	—	—
*CYB5R3*	lobular inflammation	+0.093 (0.55)	not graded	not graded	—	—	—
*MTARC1*	fibrosis stage	−0.323 (0.015)	−0.180 (0.026)	−0.243 (0.042)	−0.224 (−0.317 to −0.128)	3 × 10^−5^	0%
*MTARC1*	NAFLD activity score	−0.222 (0.10)	−0.039 (0.69)	−0.343 (0.003)	−0.193 (−0.371 to −0.001)	0.087	70%
*MTARC1*	lobular inflammation	−0.341 (0.009)	not graded	not graded	—	—	—
*MTARC1*	ballooning grade	−0.110 (0.47)	not graded	not graded	—	—	—
*MTARC1*	steatosis grade	−0.068 (0.68)	not graded	not graded	—	—	—
*PARP16*	fibrosis stage	−0.012 (0.93)	−0.187 (0.020)	−0.246 (0.042)	−0.169 (−0.264 to −0.070)	0.002	0%
*PARP16*	NAFLD activity score	+0.020 (0.93)	+0.029 (0.77)	−0.330 (0.004)	−0.094 (−0.321 to +0.143)	0.50	80%
*SCD*	fibrosis stage	+0.016 (0.93)	+0.138 (0.090)	+0.202 (0.093)	+0.131 (+0.032 to +0.228)	0.023	0%
*SCD*	NAFLD activity score	+0.122 (0.41)	+0.254 (0.001)	−0.078 (0.55)	+0.111 (−0.090 to +0.303)	0.37	72%
*NQO1*	fibrosis stage	+0.120 (0.41)	+0.446 (2 × 10^−10^)	+0.013 (0.93)	+0.211 (−0.073 to +0.463)	0.21	86%
*NQO1*	NAFLD activity score	+0.203 (0.14)	+0.393 (3 × 10^−8^)	−0.026 (0.88)	+0.204 (−0.052 to +0.434)	0.19	83%
*FASN*	fibrosis stage	−0.031 (0.88)	+0.031 (0.76)	+0.063 (0.67)	+0.027 (−0.073 to +0.126)	0.64	0%
*FASN*	NAFLD activity score	+0.166 (0.24)	+0.166 (0.042)	+0.260 (0.026)	+0.190 (+0.093 to +0.284)	<0.001	0%
*COL1A1*	fibrosis stage	+0.425 (0.001)	+0.460 (8 × 10^−11^)	+0.212 (0.076)	+0.377 (+0.220 to +0.515)	3 × 10^−5^	62%
*TGFB1*	fibrosis stage	+0.351 (0.008)	+0.091 (0.28)	+0.139 (0.27)	+0.178 (+0.025 to +0.323)	0.045	53%

## Data Availability

All data derive from the public resources cited herein: UCSC Xena (https://xenabrowser.net, accessed on 24 August 2026), GEO (https://www.ncbi.nlm.nih.gov/geo, accessed on 24 August 2026; GSE14520, GSE130970, GSE135251, GSE162694), DepMap (https://depmap.org, accessed on 24 August 2026), UALCAN (https://ualcan.path.uab.edu, accessed on 15 July 2026), KM Plotter (https://kmplot.com, accessed on 15 July 2026), cBioPortal (https://www.cbioportal.org, accessed on 15 July 2026) and the Human Protein Atlas (https://www.proteinatlas.org, accessed on 15 July 2026). The complete analysis pipeline, comprising all scripts, sample-selection criteria, preprocessing steps, model formulas, the numerical source data underlying every figure and table entry computed in this study, run logs, package versions, and session information, is available at https://github.com/drswnam69-debug/cyb5r3-marc1-lihc (accessed on 24 August 2026) and archived with the citable DOI 10.5281/zenodo.21987539. The portal-derived values quoted for comparison in Table 2 and Table 3 are labeled as portal outputs there and are not produced by the pipeline; Figure 1 and Figure 6 are schematics and contain no data. The repository was deposited before peer review of this revision and is publicly accessible.

## Supplementary Materials

The following supporting information can be downloaded at: https://www.mdpi.com/article/10.3390/ijms27177806/s1.

## Abbreviations

The following abbreviations are used in this manuscript:

MASLD	metabolic dysfunction-associated steatotic liver disease
MASH	metabolic dysfunction-associated steatohepatitis
HCC	hepatocellular carcinoma
HBV	hepatitis B virus
HCV	hepatitis C virus
LIHC	liver hepatocellular carcinoma
LUAD	lung adenocarcinoma
HR	hazard ratio
CI	confidence interval
SD	standard deviation
FDR	false-discovery rate
TCGA	The Cancer Genome Atlas
TCGA-CDR	TCGA Clinical Data Resource
GEO	Gene Expression Omnibus
GTEx	Genotype-Tissue Expression
RSEM	RNA-Seq by Expectation-Maximization
CRISPR	clustered regularly interspaced short palindromic repeats
CPTAC	Clinical Proteomic Tumor Analysis Consortium
TPM	transcripts per million
ER	endoplasmic reticulum
ROS	reactive oxygen species
NADH	reduced nicotinamide adenine dinucleotide
NAD^+^	oxidized nicotinamide adenine dinucleotide
NADPH	reduced nicotinamide adenine dinucleotide phosphate
PPP	pentose phosphate pathway
GSSG	oxidized glutathione
MUFA	monounsaturated fatty acid
SFA	saturated fatty acid
GalNAc	N-acetylgalactosamine
GAN	Gubra amylin NASH
NAFLD	non-alcoholic fatty liver disease (retained here only as the published name of the NAFLD activity score)
AJCC	American Joint Committee on Cancer
TNM	tumor–node–metastasis
IQR	interquartile range
HGNC	HUGO Gene Nomenclature Committee
RMA	robust multi-array average
AIC	Akaike information criterion
REMARK	Reporting Recommendations for Tumor Marker Prognostic Studies
STROBE	Strengthening the Reporting of Observational Studies in Epidemiology
