# Supplementary material for: Divergence of the CYB5R3–mARC1 Redox Axis Across the Human MASLD-to-Hepatocellular-Carcinoma Continuum: An Exploratory Analysis of Liver-Biopsy and Tumor Cohorts
Source: Int J Mol Sci. 2026 Aug 31;27(17):7806. doi: 10.3390/ijms27177806 (PMC13566810; doi:10.3390/ijms27177806)
Supplement: Supplementary file 1 [file ijms-27-07806-s001.zip › ijms-4481300-supplementary/CYB5R3_IJMS_R1_Supplementary_Methods.pdf]

Supplementary Methods and Tables

Manuscript: Divergence of the CYB5R3–mARC1 redox axis across the human MASLD-to-hepatocellular-carcinoma continuum: an exploratory analysis of liver-biopsy and tumor cohorts **Manuscript ID:** ijms-4481300 (revision 1)

S0. Status of the analyses and where they are reported

The original submission placed four analyses in this supplement and described them as prospectively specified extensions. In this revision they have been moved into the Results of the main text, in accordance with the reviewers' request that new results should not appear only in the Discussion or the supplement. This file therefore serves two purposes: it documents the methods that are too detailed for Section 4, and it carries the legends of the supplementary tables and figures. The full content of every table is supplied as Supplementary_Tables_S1_S7.xlsx, one worksheet per table, and is also the deposited result files named in each legend; Table S6 is short enough to be printed here in full as well.

Analysis	Reported in the main text	Full table here
Raw-data tumor-versus-normal expression, four estimates	Section 2.2, Table 2	Table S1
Primary and extended survival models	Section 2.3	Table S2
Pan-cancer scan across TCGA cohorts	Section 2.7	Table S3
Stage, grade and etiology within TCGA-LIHC	Section 2.9	Table S4
CYB5R3 redox panel: exploratory pairwise associations, liver versus lung	Section 2.6, Table 4	Table S5
DepMap CRISPR dependency	Section 2.5	Table S6
Human MASLD liver-biopsy cohorts	Section 2.8, Table 5	Table S7

All analyses were implemented in R and depend only on publicly available datasets. A single random seed is fixed, each script records the full computational environment with sessionInfo(), and all summary statistics and figures are written to a dedicated output directory. **The complete pipeline is deposited in a public repository with a citable DOI and was publicly accessible before peer review of this revision** (Data Availability Statement in the main text). The three inconsistent statements about code availability in the original submission, which promised the scripts "accompany this article", "will be deposited on publication" and constituted "a fully reproducible pipeline", have been replaced by that single statement.

S1. Raw-data expression analysis

Gene-level expression was retrieved from three UCSC Xena matrices that differ in their processing: TCGA.LIHC.sampleMap/HiSeqV2 and TCGA.LUAD.sampleMap/HiSeqV2 (TCGA RNA-seq V2, log2(norm_count+1)); TcgaTargetGtex_rsem_gene_tpm (the TOIL recompute, in which TCGA and GTEx samples pass through one uniform pipeline, log2(TPM+0.001)); and EB++AdjustPANCAN_IlluminaHiSeq_RNASeqV2.geneExp.xena (PanCanAtlas, batch-corrected, log2). On the two HiSeqV2 matrices, sample type was taken from the TCGA barcode: codes 01, 02, 03, 05, 06 and 07 were treated as tumor and 11 as adjacent normal. On the TOIL and PanCanAtlas matrices, which are indexed by phenotype, samples were selected by case-insensitive matching of the phenotype field against Primary Tumor for tumors and against Normal for the adjacent-normal reference, within TCGA samples only; this stricter rule is why the TOIL tumor count is 369 where the HiSeqV2 count is 373, and 513 where the LUAD HiSeqV2 count is 517. GTEx normal tissue was identified from TcgaTargetGTEX_phenotype.txt. The counts in this section are samples; where an analysis is at the patient level, the first sample for each patient in matrix column order was retained.

Groups were compared by the Wilcoxon rank-sum test, with the difference of medians on the log2 scale as the effect estimate and Cliff's delta as a non-parametric effect size. Linear-scale TPM values quoted in the main text were obtained as 2^x − 0.001 from the TOIL matrix.

MTARC1 could not be retrieved from either legacy HiSeqV2 probe map under any of its aliases (*MTARC1*, *MARC1*, *MOSC1*), which is one reason the TOIL recompute is preferred for that gene. The aliases queried are fixed in the shared setup file. The alias that succeeded is recorded in the alias_used column of results/10_expression_estimates.csv for the tumor-versus-normal matrices; for the TOIL retrieval used in the survival analyses it is recorded in the note column of results/11_flow.csv, and for the remaining retrievals the alias and any lookup failure appear in the deposited run log rather than in a result file.

Table S1. Complete tumor-versus-normal estimates for *CYB5R3* and *MTARC1* in LIHC and LUAD, by matrix and normal reference, with n, median, interquartile range, log2 fold change, Cliff's delta and Wilcoxon p. *(Provided in full in Supplementary_Tables_S1_S7.xlsx, sheet "S1 Tumor-vs-normal", and as results/10_expression_estimates.csv in the deposited repository.)*

S2. Survival analysis

The primary analysis is a Cox proportional-hazards model of overall survival on expression standardized to unit standard deviation, adjusted for age, sex and AJCC stage dichotomized as I–II versus III–IV, with stage IIIA–IIIC counted as III and IVA–IVB as IV. Survival data were taken from the TCGA Clinical Data Resource. *CYB5R3* is measured on the HiSeqV2 matrix and *MTARC1* on the TOIL matrix, since *MTARC1* is

absent from the legacy probe map; each gene is standardized within the analysis set of the model in which it appears, so "per standard deviation" refers to that set rather than to one cohort-wide standard deviation. Progression-free interval, also from the Clinical Data Resource, was analyzed as a sensitivity endpoint on its own complete-case set. Every term of the primary models, including age, sex and stage, is deposited in results/11_cox_primary_full_coefficients.csv.

Proportional hazards were assessed by scaled Schoenfeld residuals for each covariate and globally. A non-linear expression effect was tested by comparing the linear term with a penalized spline (3 degrees of freedom) by likelihood ratio and by AIC. Discrimination was quantified by Harrell's C-index for a clinical base model containing age, sex and stage and for that model plus expression, with the difference and its percentile confidence interval obtained from 1000 bootstrap resamples. Both models are refitted and evaluated on each resample, so the C-indices are apparent values without an optimism correction. The two axis members were modeled jointly, with a likelihood-ratio test against the single-gene model, a multiplicative interaction term, and a single term expressing the difference of the two standardized values.

Missing covariates were handled by complete-case analysis for the primary model. Multiple imputation by chained equations (m = 20, including the Nelson–Aalen cumulative hazard as an auxiliary variable) was pre-specified as a sensitivity analysis and gave the same point estimate with slightly greater precision (n = 363, 128 deaths; HR 1.23 per SD, 95% CI 1.03–1.48, p = 0.026).

In GSE14520 the two Affymetrix series matrices were combined as deposited, without cross-platform normalization and without a platform term; serum α-fetoprotein was specified for the extended model there but is recorded as a single category and was dropped automatically, so that model contains age, sex, TNM stage, cirrhosis and multinodularity only. In TCGA-LIHC, extended-covariate models used the measured α-fetoprotein field (fetoprotein_outcome_value, available for 261 of 339 patients), the vascular-invasion field and the Ishak fibrosis score dichotomized at stage 5. Note that the TCGA-LIHC clinical matrix also contains fetoprotein_outcome_lower_limit and ..._upper_limit, which record assay limits rather than concentrations and must not be used as the covariate.

For GSE14520, the series matrices were used as deposited (RMA-normalized, log2), all probe sets annotated to each gene symbol were averaged, and the endpoint was overall survival in months with death from any cause as the event. The probe identifiers used are deposited as results/11_gse14520_probe_selection.csv.

Table S2. Primary, secondary, sensitivity, joint, interaction and extended-covariate survival models, with n, events, hazard ratios, confidence intervals and p-values; proportional-hazards diagnostics; non-linearity test; C-index and change in C-index. *(Provided in full in Supplementary_Tables_S1_S7.xlsx, sheet "S2 Survival models", assembled from results/11_*.csv and results/17_figS7_ph_test.csv.)*

S3. Pan-cancer scan (TCGA)

CYB5R3 was surveyed across TCGA cohorts using the PanCanAtlas hub. Batch-corrected, gene-level expression was retrieved as a single-gene slice, avoiding download of the full matrix, and curated overall-survival data were taken from the TCGA Clinical Data Resource. Because the survival table is keyed by 12-character patient barcodes whereas expression columns are sample barcodes, records were joined on the patient barcode after restricting expression to primary tumors with one sample per patient.

For each cohort with at least 20 evaluable tumors and at least 10 deaths, expression was standardized within the cohort and related to overall survival by univariable Cox regression. Tumor-versus-normal expression was compared in cohorts with at least 10 adjacent-normal samples by the Wilcoxon rank-sum test. **Benjamini–Hochberg correction was applied across cohorts, separately for the survival family and the expression family of tests.**

Twenty-eight cohorts met the inclusion criteria. **After Benjamini–Hochberg correction across cohorts, no cohort retained a significant survival association, including LIHC** (nominal HR 1.20 per SD, 95% CI 1.01–1.44, p = 0.044, q = 0.19; the smallest corrected value anywhere in the survival family is q = 0.146).

Table S3. *CYB5R3* prognostic direction across all 28 evaluable TCGA cohorts, with tumor-versus-normal expression in the 15 of them that have at least 10 adjacent-normal samples, with hazard ratio per standard deviation, 95% confidence interval, p-value and Benjamini–Hochberg q-value, and log2 fold change with its p and q. *(Provided in full in Supplementary_Tables_S1_S7.xlsx, sheet "S3 Pan-cancer", and as results/13_pancancer_full.csv in the deposited repository.)*

> **Caption note, corrected from the original submission.** The LIHC row of this table draws on **the same TCGA-LIHC patient population as the primary analysis in Section 2.3** and is therefore **not an independent reproduction of it**; it is not the identical set, because it is selected on the PanCanAtlas matrix by a different route and contains 365 patients against the 339 of the primary analysis; the original caption's description of it as "matching the main analysis" has been removed. The LIHC tumor-versus-normal estimate on this batch-corrected matrix is **slightly lower in tumor and not significant** (log2 fold change −0.06, p = 0.104 in the 365 patients this table uses; the same contrast in Table 2 of the main text gives p = 0.102 in 369 patients), which points in the opposite direction from the UALCAN rendering and from the TOIL recompute. The original caption stated that this table, together with the LUAD row, reproduced the liver–lung contrast; that statement was incorrect for the liver side and has been withdrawn. Section 2.2 and Table 2 of the main text now present all four estimates side by side and explain that the direction of the liver contrast depends on the processing pipeline, which is why the manuscript no longer claims that *CYB5R3* is over-expressed in HCC.

S4. Stage-, grade- and etiology-stratified analysis in TCGA-LIHC

Gene-level expression and the matched clinical matrix were obtained from UCSC Xena, and primary-tumor samples were retained. Tumor stage was harmonized to AJCC groups I–IV and histologic grade to G1–G4. Etiology was classified from the viral_hepatitis_serology field as viral serology positive (field populated) versus no positive viral serology recorded (field empty).

> **Correction from the original submission.** This group was previously labeled the "non-viral/metabolic" subset. On re-examination the TCGA-LIHC viral_hepatitis_serology field **lists the serologies that returned a positive result** and is populated for 164 of 371 primary tumors; an empty field means no positive serology was recorded and cannot be distinguished from a patient who was never tested. The original subset was constructed on the opposite reading of this field. **No metabolic subset can be defined from these data**, the estimate derived from the original subset is withdrawn, and Section 3.5 of the main text lists the absence of any metabolically defined HCC population as the study's most important limitation.

Expression across ordered stage and grade categories was tested for a monotone trend by the Jonckheere–Terpstra test (package clinfun; normal approximation, since the sample exceeds 100 and contains ties), with Kruskal–Wallis as a fallback. Regenerating this analysis from raw data changed the values reported in the original submission: the stage trend is p = 0.164 (previously 0.283) and the grade trend p = 0.007 (previously 0.0019). The regenerated values are the ones reported. The two etiology groups were compared by the Wilcoxon rank-sum test. Within each group the prognostic value of *CYB5R3* was assessed by Cox regression on standardized expression, and the estimates are exploratory. **Neither group corresponds to a metabolically defined population, and the manuscript does not present either as one.**

The stage trend was not significant (Jonckheere–Terpstra p = 0.164) and expression showed a decreasing trend across histologic grade (p = 0.007). Of 371 primary tumors, 164 had at least one positive viral serology recorded and 207 had none; expression did not differ between them (Wilcoxon p = 0.209).

Table S4. *CYB5R3* in TCGA-LIHC by stage, histologic grade and viral-serology status, with group sizes, medians and trend-test p-values, and the exploratory survival estimates in each serology group. *(Provided in full in Supplementary_Tables_S1_S7.xlsx, sheet "S4 Stage grade etiology", and as results/16_lihc_stage_grade.csv, results/16_lihc_etiology.csv and results/16_lihc_serologynegative_cox.csv in the deposited repository.)*

S5. CYB5R3 redox panel: exploratory pairwise associations, liver versus lung

Gene-level RNA-seq for *CYB5R3* and a pre-specified 16-gene redox panel (*SCD*, *PARP16*, *NQO1*, *NFE2L2*, *KEAP1*, *GCLC*, *GCLM*, *GSR*, *GPX4*, *TXN*, *TXNRD1*, *SOD1*, *SOD2*, *CAT*, *PRDX1*, *G6PD*) was obtained for TCGA-LIHC and TCGA-LUAD. Within primary tumors of each cohort, Spearman correlations of *CYB5R3* with each panel gene were computed with Fisher-z 95% confidence intervals and Benjamini–Hochberg q-values. Fisher-z variances were computed as $1/(n - 3)$, the Pearson expression, without a Spearman-specific correction, so the intervals are marginally narrower and the z statistics marginally larger than a Spearman-specific variance would give.

Three analyses were added in this revision.

Formal comparison between tissues. The liver and lung coefficients were compared gene by gene by Fisher's r-to-z transformation, with the difference and its 95% confidence interval, and Benjamini–Hochberg correction across the panel.

Adjustment for tumor cellular composition. Partial Spearman correlations were computed on rank-transformed variables. The pipeline uses the ABSOLUTE consensus purity call only when at least 100 tumors of a cohort carry one, and falls back to a marker-based proxy otherwise. The TCGA ABSOLUTE consensus purity table could not be resolved on the UCSC Xena hubs under the identifier queried, and no purity call could be matched to any sample in either cohort, so the conditioning variable was the first principal component of a panel of established stromal and immune marker genes (*COL1A1*, *COL1A2*, *COL3A1*, *FN1*, *ACTA2*, *PDGFRB*, *THY1*, *LUM*, *PTPRC*, *CD3E*, *CD2*, *CD8A*, *CD68*, *CSF1R*, *ITGAM*, *LYZ*, *HLA-DRA*), with each marker standardized to zero mean and unit variance and any missing value set to that marker's own mean and the component sign flipped so that higher values are intended to indicate higher tumor content; partial correlations are invariant to that sign, so the orientation affects interpretation of the covariate only and not any reported coefficient. This is a weaker adjustment than a purity call and is described as such wherever it is used. The composition-adjusted coefficients are corrected in their own Benjamini–Hochberg families, one of 16 tests within each cohort and one of 16 for the adjusted tissue-difference comparison.

Permutation null. *CYB5R3* expression was permuted within each cohort 10,000 times and the number of sign reversals across the panel was recorded, to test whether the observed count exceeds chance.

Table S5. Complete 16-gene panel in TCGA-LIHC and TCGA-LUAD, with Spearman coefficient, 95% confidence interval, p-value and Benjamini–Hochberg q-value in each cohort; the Fisher r-to-z statistic, its p and q; the composition-adjusted coefficients; the adjusted tissue-difference test; and the permutation null for the number of sign reversals. *(Provided in full in Supplementary_Tables_S1_S7.xlsx, sheet "S5 Co-expression panel", and as results/12_coexpression_full.csv, results/12_tissue_difference.csv, results/12_purity_adjusted.csv,*

results/12_tissue_difference_purity_adjusted.csv and results/12_permutation_reversals.csv in the deposited repository.)

> **Caption note, corrected from the original submission.** The original caption described a tissue-inverted redox **module**. That term overstates what a small set of pre-selected genes with weak correlations can support, and it has been replaced throughout by **exploratory pairwise associations**. The corrected reading is as follows. Within liver, **only *SCD* survives false-discovery-rate correction** (r_s = +0.164, q = 0.024); *PARP16* (−0.131, q = 0.086) and *NQO1* (+0.114, q = 0.090) do not. The **number** of sign reversals across the panel is exactly what permutation yields (8 observed against a permutation mean of 8.0, p = 0.58), so no general inversion is demonstrated. What is demonstrated is narrower: **six specific coefficients differ significantly between the two tissues after correction** (*CAT*, *PARP16*, *TXNRD1*, *SOD2*, *SCD*, *KEAP1*), and all six survive adjustment for tumor cellular composition.

S6. DepMap CRISPR dependency

Genome-wide CRISPR–Cas9 knockout screens were obtained from the Cancer Dependency Map through the Bioconductor depmap package (version 1.26.0; the ExperimentHub release is resolved at run time rather than pinned in the script, and the deposited run resolved to the snapshot of 2026-04-21, which the package reports at load time and which is therefore recorded in the deposited run log rather than in a result file); a manual route using CRISPRGeneEffect.csv and Model.csv from the DepMap portal is provided as an alternative. Cell lines were annotated by DepMap lineage and grouped as liver or lung. The grouping is the lineage annotation, not the TCGA histology used in Sections 2.2 and 2.6, so the lung set is broader than LUAD. Chronos scores were interpreted on the standard scale, on which 0 indicates no effect, −1 approximates the median effect of common essential genes, and values below −0.5 denote a dependent line. The two lineages were compared by a two-sided Wilcoxon rank-sum test.

A null or reversed result would count against the tumor-dependency premise, and that is what was obtained. The study is exploratory and this comparison was not pre-registered.

Table S6. *CYB5R3* dependency by lineage (DepMap CRISPR/Chronos). *(Also in Supplementary_Tables_S1_S7.xlsx, sheet "S6 DepMap", and deposited as results/15_depmap_dependency.csv.)*

Lineage	n lines	Median gene effect	IQR (Q1, Q3)	% lines < −0.5	Wilcoxon p
Liver	22	−0.046	(−0.078, +0.002)	0%	0.165
Lung	133	−0.018	(−0.065, +0.023)	0.75%	0.165

Median difference, liver minus lung: −0.028.

Correction from the original submission. The original main text and supplement quoted this comparison as p = 0.17 and p = 0.165 respectively. A single value is now produced by one deposited script and used everywhere: **p = 0.165**.

S7. Human MASLD liver-biopsy cohorts

Hepatic expression of *CYB5R3*, *MTARC1*, *SCD* and *NQO1*, together with *PARP16*, *FASN* and the fibrogenic positive controls *COL1A1* and *TGFB1*, was examined in GSE130970 (78 biopsies with pathologist-assigned grades for steatosis 0–3, lobular inflammation 0–2, cytological ballooning 0–2, NAFLD activity score 0–7 and fibrosis stage 0–4), GSE135251 (216 biopsies with NAFLD activity score and fibrosis stage) and GSE162694 (143 biopsies, of which 128 could be matched to their sample annotation; among the matched samples 99 carried a fibrosis stage and 102 a NAFLD activity score in the deposited annotation, and only samples with a recorded grade enter the corresponding test). Sample identifiers in GSE162694 do not correspond to GEO accessions, so the pipeline scores every candidate annotation column by normalized exact match and then by bidirectional substring containment, adopts the best-scoring column as the key and requires the match to be unique; on the deposited run the sample title was selected, at 89.5% coverage.

Deposited count matrices were converted to counts per million where they were not already normalized, and values were log2(x+1)-transformed. Gene identifiers were resolved across symbol, Entrez and Ensembl conventions; where a symbol maps to more than one row of a deposited matrix the first matching row was used rather than an average, which differs from the probe averaging used for GSE14520. Associations with ordinal histological grades were tested by Spearman rank correlation, with **Benjamini–Hochberg correction applied across all 72 gene × histology × cohort tests**. Kruskal–Wallis statistics were computed alongside them and are reported uncorrected in the deposited output; they are not part of the corrected family. Two further correction families are kept separate from it: the 16 pooled meta-analytic tests (eight genes × two pooled endpoints), which supply the pooled q of Table 5, and the 48 tests of the GSE130970 lesion model and its partial correlations. To separate the histological components, a linear model containing steatosis, lobular inflammation and ballooning, each entered as a numeric score, was fitted in GSE130970, the only cohort grading the three lesions separately, together with partial Spearman correlations adjusting for the remaining two lesions. Cohort estimates were pooled by random-effects meta-analysis (restricted maximum likelihood) of Fisher-z-transformed coefficients, with I^2 reported. The cohort-specific estimates are shown alongside every pooled value, because for *CYB5R3* the pooled estimate conceals a disagreement in sign between cohorts that is the substantive finding.

Table S7. Complete gene × histology × cohort results, with n, number of grade levels, Spearman coefficient, trend p, Kruskal–Wallis p and Benjamini–Hochberg q;

the multivariable and partial-correlation results in GSE130970; and the pooled estimates with confidence intervals. *(Provided in full in Supplementary_Tables_S1_S7.xlsx, sheet "S7 MASLD biopsies", and as results/14_masld_trend_all.csv, results/14_masld_partial.csv and results/14_masld_meta.csv in the deposited repository.)*

S8. Data and code availability

All analyses draw exclusively on public resources: UCSC Xena (TCGA, GTEx and PanCanAtlas hubs), the TCGA Clinical Data Resource, GEO (GSE14520, GSE130970, GSE135251, GSE162694) and the Cancer Dependency Map. No raw patient data are redistributed; every input is downloaded by the scripts from its originating repository.

The analysis scripts, a README specifying the required packages and the execution order, the session-information files that record the package versions actually used, the numerical source data underlying every figure and table entry computed in this study, the run logs and the session information are deposited in a public, versioned repository archived with a citable DOI, as stated in the Data Availability Statement of the main text. The portal-derived values quoted for comparison in Tables 2 and 3 of the main text are labeled as portal outputs there and are not produced by the pipeline; Figures 1 and 4 are schematics and contain no data. Portal values were retrieved on 15 July 2026; the raw-data analyses carry the run dates recorded in the deposited logs; the DepMap ExperimentHub release is resolved at run time rather than pinned in the script; the deposited run resolved to the snapshot of 2026-04-21.

Supplementary Figures

Figure S1. *CYB5R3* CRISPR dependency (DepMap Chronos gene effect) in liver versus lung cancer cell lines, shown as the lineage median with the interquartile range. Neither lineage's median approaches the −0.5 dependency threshold, and the difference between lineages is not significant; no liver line and one of 133 lung lines (0.75%) fall below it (Wilcoxon $p = 0.165$).

Figure S2. Per-cohort overall-survival hazard ratio for *CYB5R3* (per standard deviation) across TCGA cancers, with Benjamini–Hochberg q-values shown; no cohort is significant after correction. The LIHC point draws on the same TCGA-LIHC patient population as the primary analysis — not the identical set, since it is selected on the PanCanAtlas matrix by a different route — and is drawn in orange for that reason; it is not an independent replication.

Figure S3. Tumor-versus-normal log2 fold change plotted against log2 hazard ratio for *CYB5R3* across the 15 TCGA cohorts with at least 10 adjacent-normal samples, with LIHC drawn in orange. The association between the two quantities is positive

but not significant (Spearman r_s = 0.26, p = 0.35), so no relationship is detectable and the up-and-adverse, down-and-protective rule is not supported beyond the liver–lung contrast; with only 15 cohorts this is an absence of evidence rather than evidence of absence.

Figure S4. Median *CYB5R3* expression in TCGA-LIHC by AJCC stage, histologic grade and viral-serology status, with the group size beneath each point and the trend or comparison test in each panel heading. Expression shows a decreasing trend across histologic grade, although the group medians are not strictly monotone; stage and serology status show no significant difference. “None recorded” is not equivalent to non-viral etiology (Section 2.9).

Figure S5. *CYB5R3* pairwise associations with the redox panel in TCGA-LIHC (n = 371) and TCGA-LUAD (n = 515), with the composition-adjusted coefficients shown as open symbols alongside the unadjusted ones. Confidence intervals for the unadjusted coefficients are in Table S5; the composition-adjusted values are point estimates and have no interval in the deposited output. Magnitudes are weak; the interpretable result is the formal tissue-difference test in Table S5, not the pattern of signs.

Figure S6. Patient flow from 423 TCGA-LIHC samples with *CYB5R3* expression to the 339 patients of the primary analysis set. The first box counts samples and every later box counts patients; each box states the criterion applied, and the figure beside an arrow is the number excluded at that step. The number of deaths is given in the final box. Of the 371 patients, 365 have overall-survival data with 130 deaths, and that set is the one used in Section 2.9. The pan-cancer row in Section 2.7 has the same size but is selected on the PanCanAtlas matrix and is not the same set.

Figure S7. Scaled Schoenfeld residuals for each term of the primary *CYB5R3* Cox model, plotted against follow-up time on the Kaplan–Meier transformed scale that cox.zph uses by default, so the axis is labeled in days but is not linear in them, with the per-term p-value in each panel heading and the global test, the analysis-set size and the number of deaths in the figure heading. In each panel the solid line is the smoothed estimate of the time-varying coefficient with its approximate two-standard-error band as dashed lines; the horizontal dashed line marks zero, the no-effect reference; under proportional hazards the smoothed estimate is flat, though not necessarily at zero.
