# Supplementary material for: Divergence of the CYB5R3–mARC1 Redox Axis Across the Human MASLD-to-Hepatocellular-Carcinoma Continuum: An Exploratory Analysis of Liver-Biopsy and Tumor Cohorts
Source: Int J Mol Sci. 2026 Aug 31;27(17):7806. doi: 10.3390/ijms27177806 (PMC13566810; doi:10.3390/ijms27177806)
Supplement: Supplementary file 1 [file ijms-27-07806-s001.zip › ijms-4481300-supplementary/Supplementary_File_S1_REMARK.pdf]

Supplementary File S1 — Completed REMARK checklist

Manuscript: Divergence of the CYB5R3–mARC1 redox axis across the human MASLD-to-hepatocellular-carcinoma continuum: an exploratory analysis of liver-biopsy and tumor cohorts **Manuscript ID:** ijms-4481300 (revision 1) **Author:** Soon Woo Nam

REMARK: McShane LM, Altman DG, Sauerbrei W, Taube SE, Gion M, Clark GM. Reporting recommendations for tumor marker prognostic studies (REMARK). *J Natl Cancer Inst* 2005;97:1180–1184. doi:10.1093/jnci/dji237.

Scope note. This is a secondary analysis of public transcriptomic and clinical data, not a prospective marker study on archived specimens. Items about biological material and assay methods are answered from the originating consortia's published protocols, which is the only access this study has to them. An item that cannot be answered at all from the harmonized public resources is marked *not applicable* with the reason given, rather than left blank; item 3 is the only such item.

Introduction

#	Item	Where addressed
1	State the marker examined, the study objectives and any pre-specified hypotheses.	Section 1, final three paragraphs. Markers: *CYB5R3* and *MTARC1* transcript expression. Objectives: (i) whether the two members of the axis carry the same or divergent prognostic signal in HCC; (ii) where the axis sits in the pre-malignant human liver. The corollary tested — that endogenous *CYB5R3* becomes deficient as MASLD advances — is set out in Section 1 as an assumption of the existing literature rather than as the author's own prior belief, and it was the declared target of the liver-biopsy analysis. The study was not pre-registered. The corollary was **found to be unsupported**, since three liver-biopsy cohorts disagree on the sign of the association (Section 2.8).

Materials and methods — Patients

#	Item	Where addressed
2	Describe the characteristics (e.g., disease stage or co-morbidities) of the study patients, including their	Sections 2.3, 2.4, 4.2. TCGA-LIHC primary tumors (UCSC Xena); GEO GSE14520 (HBV-related HCC, resected, Chinese cohort). Treatment data are not available in the harmonized resources used, and

#	Item	Where addressed
	source and inclusion and exclusion criteria.	this is stated as a limitation (Section 3.5).
3	Describe treatments received and how chosen (e.g., randomized or rule-based).	**Not applicable / not available.** Neither TCGA-LIHC via UCSC Xena nor GSE14520 supplies harmonized systemic-therapy data. Stated in Section 3.5.

Materials and methods — Specimen characteristics

#	Item	Where addressed
4	Describe type of biological material used (including control samples), and methods of preservation and storage.	Section 4.2. Publicly deposited RNA-sequencing (TCGA, GTEx, liver-biopsy cohorts) and microarray (GSE14520) data. Primary specimen handling was performed by the originating consortia and is described in their primary publications: TCGA, GTEx and the TCGA Clinical Data Resource in Section 4.2 [36–38], GSE14520 in Section 2.4 [15], and the three liver-biopsy series in Sections 2.8 and 4.7 [17–19]. The four cohort papers are also listed in the repository README.

Materials and methods — Assay methods

#	Item	Where addressed
5	Specify the assay method used and provide (or reference) a detailed protocol, including specific reagents or kits used, quality control procedures, reproducibility assessments, quantitation methods, and scoring and reporting protocols. Specify whether and how assays were performed blinded to the study endpoint.	Sections 2.4, 4.2, 4.4, 4.9. Assays: Illumina HiSeq RNA-seq processed by three named pipelines (TCGA RNA-seq V2 HiSeqV2, UCSC TOIL RSEM recompute, PanCanAtlas EB++ batch-corrected) and Affymetrix HG-U133A 2.0/HT_HG-U133A for GSE14520. The probe-selection rule and preprocessing for GSE14520 are specified in Section 2.4, and the probe identifiers are deposited. Expression measurement preceded and was independent of endpoint ascertainment in every cohort, so blinding is intrinsic to the design.

Materials and methods — Study design

#	Item	Where addressed
6	State the method of case selection, including whether prospective or retrospective and whether stratification or	Sections 2.3, 4.4, 4.5. Retrospective analysis of consecutively deposited public cohorts, with no stratification or matching. Selection: tumor samples by TCGA sample-type code (01, 02, 03, 05, 06, 07; overwhelmingly code 01, primary solid tumor — Section 4.4), one sample per patient. Accrual periods are those of the originating consortia. Median follow-up 816 days by reverse Kaplan–Meier (Section 2.3). The harmonized

#	Item	Where addressed
	matching (e.g., by stage or age) was employed. Specify the time period from which cases were taken, the end of the follow-up period, and the median follow-up time.	public resources record follow-up as a duration from diagnosis, not as calendar dates, so neither the accrual window nor the calendar end of follow-up can be recovered from them; this part of the item cannot be met from the data used and is declared here rather than left blank.
7	Precisely define all clinical endpoints examined.	Section 4.5. Primary endpoint: overall survival (death from any cause) from the TCGA Clinical Data Resource. Secondary: progression-free interval (TCGA-CDR definition). GSE14520: overall survival in months, death from any cause.
8	List all candidate variables initially examined or considered for inclusion in models.	Sections 2.3, 4.5. Age, sex, AJCC stage (I–II versus III–IV), plus, where available, α-fetoprotein, vascular invasion and cirrhosis (Ishak 5–6). Availability and completeness of each field are reported in results/11_extended_covariate_availability.csv. Histologic grade and viral serology were examined as stratifiers (Section 2.9), not as model covariates.
9	Give rationale for sample size; if the study was designed to detect a specified effect size, give the target power and effect size.	Section 3.5. **No formal sample-size calculation.** Cohort sizes were fixed by the public resources. This is a limitation, stated explicitly. The subgroup analysis that was underpowered in the original submission has been withdrawn altogether, because the field used to define it was misread (Section 2.9).

Materials and methods — Statistical analysis methods

#	Item	Where addressed
10	Specify all statistical methods, including details of any variable selection procedures and other model-building issues, how model assumptions were verified, and how missing data were handled.	Sections 4.4–4.6, 4.8. No variable-selection procedure was used; the primary model was pre-specified. Proportional hazards verified by scaled Schoenfeld residuals per term and globally (Section 2.3, Supplementary Figure S7). Linearity verified against a penalized spline. Missing covariates handled by complete-case analysis with multiple imputation (m = 20, Nelson–Aalen cumulative hazard auxiliary) as sensitivity analysis.
11	Clarify how marker	Sections 2.3, 4.5. **The marker was analyzed as a**

#	Item	Where addressed
	values were handled in the analyses; if relevant, describe methods used for cutpoint determination.	**continuous variable standardized to unit standard deviation, and this is designated the primary analysis.** Median-split and portal optimal-cut-off analyses are reported as secondary and labeled as such, with the reason (a data-derived cut-off inflates apparent significance).

Results — Data

#	Item	Where addressed
12	Describe the flow of patients through the study, including the number of patients included in each stage of the analysis (a diagram may be helpful) and reasons for dropout. Specifically, both overall and for each subgroup extensively examined report the number of patients and the number of events.	Section 2.3 and **Supplementary Figure S6**. 423 samples with expression → 371 primary tumors (one per patient) → 369 matched to *MTARC1* → 363 with non-missing survival → 339 with complete covariates, **114 deaths**. Progression-free interval: 162 events. GSE14520: n = 242, 96 deaths. Extended-covariate subsets: α-fetoprotein n = 261 (74 deaths), vascular invasion n = 291 (85 deaths), cirrhosis n = 196 (58 deaths), vascular invasion and cirrhosis together n = 188 (54 deaths). Etiology subgroups examined for survival: no positive viral serology recorded n = 203 with 59 deaths, viral serology positive n = 162 with 71 deaths; expression compared in 164 versus 207 primary tumors; neither is a metabolically defined population (Section 2.9).
13	Report distributions of basic demographic characteristics (at least age and sex), standard (disease-specific) prognostic variables, and tumor marker, including numbers of missing values.	Section 2.3 and results/11_missing_compare.csv; expression distributions with median, interquartile range and n in **Figure 2** and results/10_expression_estimates.csv. Included and excluded patients are compared for age, sex, marker and survival; stage cannot be compared because missing stage is itself an exclusion criterion. No separate demographic table is presented, so this item is addressed in part.

Results — Analysis and presentation

#	Item	Where addressed
14	Show the relation of the marker to standard prognostic variables.	Section 2.9 (stage, histologic grade, viral serology) and Section 2.3 (extended-covariate models). Notably, marker expression shows a **decreasing trend** across histologic grade, which is reported rather than omitted.
15	Present univariate	Sections 2.4, 2.7, 2.9 and **Figure 6**. Univariable estimates:

#	Item	Where addressed
	analyses showing the relation between the marker and outcome, with the estimated effect (e.g., hazard ratio and survival probability). Preferably provide similar analyses for all other variables being analyzed. For the effect of a tumor marker on a time-to-event outcome, a Kaplan-Meier plot is recommended.	the TCGA-LIHC univariable estimate in Section 2.7 (HR 1.20 per SD, 95% CI 1.01–1.44), the external cohort unadjusted in Section 2.4 (*CYB5R3* HR 1.42, 95% CI 1.14–1.76; *MTARC1* HR 0.73, 95% CI 0.60–0.89) and univariable estimates for all 28 TCGA cohorts in results/13_pancancer_full.csv. Multivariable estimates are reported under item 16. Univariable analyses of the other model variables are not presented separately; their coefficients are in results/11_cox_primary_full_coefficients.csv. **No Kaplan–Meier plot is provided**: the marker is analyzed as a continuous variable throughout, and the only dichotomized estimates in the manuscript are portal outputs (Table 3), which are labeled as such.
16	For key multivariable analyses, report estimated effects (e.g., hazard ratio) with confidence intervals for the marker and, at least for the final model, all other variables in the model.	Section 2.3 and results/11_cox_primary_full_coefficients.csv, which reports every term of the two primary models — marker, age, sex and stage — with its hazard ratio, 95% confidence interval and p-value, the marker term being deposited for the secondary, joint, interaction, extended-covariate and external-cohort models in their own results files; the marker coefficients are also given in Section 2.3 and **Supplementary Table S2**.
17	Among reported results, provide estimated effects with confidence intervals from an analysis in which the marker and standard prognostic variables are included, regardless of their statistical significance.	Sections 2.3 and 2.4, and results/11_cox_primary.csv, results/11_cox_joint.csv, results/11_cox_extended.csv. Non-significant estimates (*MTARC1* within TCGA, both progression-free-interval models, the joint and interaction models, every extended-covariate model) are reported with their confidence intervals.
18	If done, report results of further investigations, such as checking assumptions, sensitivity analyses, and internal validation.	Sections 2.3 and 2.6. Proportional-hazards diagnostics; spline check for non-linearity; bootstrap C-index and ΔC-index (1000 resamples); multiple-imputation sensitivity analysis; joint and interaction models; extended-covariate models; Fisher r-to-z testing with FDR correction; adjustment for tumor cellular composition; 10,000-permutation null.

Discussion

#	Item	Where addressed
19	Interpret the results in the context of the pre-specified hypotheses and other relevant studies; include a discussion of limitations of the study.	Sections 3.1–3.5. The study was not pre-registered and states no prior hypothesis of its own (item 1); the corollary it set out to test, drawn from the existing literature, was left unsupported — neither confirmed nor refuted — and this is stated. Section 3.4 places the findings against the seven in vivo mARC1 studies, including the two whose results contradict the others.
20	Discuss implications for future research and clinical value.	Section 3.6. Falsifiable predictions only. **No clinical recommendation is made**, and the therapeutic proposals of the original submission are withdrawn (Sections 3.3, 3.4).

Summary of REMARK compliance. Items 1, 2, 4, 5, 7, 8, 10–12, 14, 16–20 are addressed in full. Item 6 is addressed except for the accrual window and the calendar end of follow-up, which the harmonized public resources do not record. Item 13 is addressed in part (no separate demographic table). Item 15 is addressed in part: univariable marker estimates and the deposited coefficients of the other model variables are given, but the other variables are not presented as separate univariable analyses and no Kaplan–Meier plot is provided, because the marker is analyzed as a continuous variable throughout. Item 3 (treatment received) is not available in the harmonized public resources and is declared as a limitation. Item 9 (sample-size rationale) is declared as absent, since cohort sizes were fixed by the public resources; the manuscript reports the resulting imprecision rather than a power calculation.
