# Supplementary material for: Divergence of the CYB5R3–mARC1 Redox Axis Across the Human MASLD-to-Hepatocellular-Carcinoma Continuum: An Exploratory Analysis of Liver-Biopsy and Tumor Cohorts
Source: Int J Mol Sci. 2026 Aug 31;27(17):7806. doi: 10.3390/ijms27177806 (PMC13566810; doi:10.3390/ijms27177806)
Supplement: Supplementary file 1 [file ijms-27-07806-s001.zip › ijms-4481300-supplementary/Supplementary_File_S2_STROBE.pdf]

Supplementary File S2 — Completed STROBE checklist (cohort studies)

Manuscript: Divergence of the CYB5R3–mARC1 redox axis across the human MASLD-to-hepatocellular-carcinoma continuum: an exploratory analysis of liver-biopsy and tumor cohorts **Manuscript ID:** ijms-4481300 (revision 1) **Author:** Soon Woo Nam

STROBE: von Elm E, Altman DG, Egger M, Pocock SJ, Gøtzsche PC, Vandenbroucke JP. The Strengthening the Reporting of Observational Studies in Epidemiology (STROBE) statement. *Lancet* 2007;370:1453–1457. doi:10.1016/S0140-6736(07)61602-X.

Scope note. This is a secondary analysis of de-identified public cohorts. Items requiring contact with participants or access to primary records are marked accordingly.

#	Item	Recommendation	Where addressed
1a	Title and abstract	Indicate the study's design with a commonly used term in the title or the abstract	Title states "exploratory analysis of liver-biopsy and tumor cohorts"; Abstract states that the observations are associative and hypothesis-generating
1b	Title and abstract	Provide in the abstract an informative and balanced summary of what was done and what was found	Abstract reports the primary estimate together with the ΔC-index showing minimal added discrimination, the absence of correlation between the two transcripts, and the failure of the MASLD estimates to replicate
2	Introduction — background/rationale	Explain the scientific background and rationale for the investigation being reported	Section 1
3	Introduction — objectives	State specific objectives, including any prespecified hypotheses	Section 1, final three paragraphs. The corollary tested — hepatic *CYB5R3* deficiency in advancing MASLD — is set out in Section 1 as an assumption of the existing literature and was the declared target of the biopsy analysis; the study was not preregistered. It was found to be unsupported
4	Metho	Present key elements	Sections 2.1 and 4.1. Section 2.1 and Table 1

#	Item	Recommendation	Where addressed
	ds — study design	of study design early in the paper	separate portal-derived observations from analyses performed here
5	Methods — setting	Describe the setting, locations, and relevant dates, including periods of recruitment, exposure, follow-up, and data collection	Section 4.2. Portal values retrieved 15 July 2026; raw-data analyses carry the run dates recorded in the deposited logs; the DepMap ExperimentHub release is resolved at run time rather than pinned in the script, and the deposited run resolved to the snapshot of 2026-04-21. Recruitment periods are those of the originating consortia. The harmonized public resources record follow-up as a duration from diagnosis rather than as calendar dates, so neither the recruitment window nor the calendar end of follow-up can be recovered from them; this part of the item cannot be met from the data used and is declared here rather than left blank
6a	Methods — participants	Give the eligibility criteria, and the sources and methods of selection of participants. Describe methods of follow-up	Sections 4.4 and 4.7. Primary tumors by TCGA sample-type code, one sample per patient; liver-biopsy cohorts as deposited. Follow-up as recorded in the TCGA Clinical Data Resource and the GSE14520 supplementary table
6b	Methods — participants	For matched studies, give matching criteria and number of exposed and unexposed	Not applicable; no matching
7	Methods — variables	Clearly define all outcomes, exposures, predictors, potential confounders, and effect modifiers. Give diagnostic criteria, if applicable	Sections 4.4, 4.5, 4.7. Exposure: standardized transcript expression. Outcome: overall survival; secondary progression-free interval; in the biopsy cohorts, pathologist-assigned histological grade. Confounders: age, sex, AJCC stage, and where available α-fetoprotein, vascular invasion and cirrhosis. Histological grades are the pathologist-assigned NAFLD activity scores and fibrosis stages recorded by the originating cohorts (Section 4.7); the scoring systems differ between cohorts, and this is stated as a limitation in Section 3.5
8	Methods —	For each variable of interest, give sources	Section 4.2 lists every dataset identifier and its normalization. Section 4.4 states explicitly that

#	Item	Recommendation	Where addressed
	data sources/measurement	of data and details of methods of assessment (measurement). Describe comparability of assessment methods if there is more than one group	three matrices with different normalizations and different normal references give different answers, which is why all are reported (Table 2)
9	Methods — bias	Describe any efforts to address potential sources of bias	Sections 2.2, 2.6, 4.4–4.6. Pipeline-dependence addressed by reporting all four estimates; cut-point bias addressed by pre-designating the continuous model as primary; tumor cellular composition addressed by partial correlation; multiplicity addressed by Benjamini–Hochberg correction within each pre-specified family of exploratory tests (Sections 4.6, 4.7, 4.8); the Kruskal–Wallis statistics and the stratified analyses of Section 2.9 are reported uncorrected and labeled exploratory; resource non-independence addressed explicitly in Section 2.1
10	Methods — study size	Explain how the study size was arrived at	Section 3.5. Cohort sizes were fixed by the public resources; no sample-size calculation was performed, and this is declared
11	Methods — quantitative variables	Explain how quantitative variables were handled in the analyses. If applicable, describe which groupings were chosen and why	Sections 4.4, 4.5. Expression analyzed continuously, standardized to unit standard deviation. Stage grouped as AJCC I–II versus III–IV with the encoding of substages stated. Median split reported only as a secondary analysis, with the reason
12a	Methods — statistical methods	Describe all statistical methods, including those used to control for confounding	Sections 4.4–4.8
12b	Methods — statistical metho	Describe any methods used to examine subgroups and interactions	Sections 2.3, 2.9, 4.5, 4.8. Joint and multiplicative-interaction models for the two genes; stratification by stage, histologic grade and viral serology; the etiology groups are reported as exploratory, and the manuscript

#	Item	Recommendation	Where addressed
	ds		states that neither corresponds to a metabolically defined population
12c	Methods — statistical methods	Explain how missing data were addressed	Sections 2.3, 4.5. Complete-case primary analysis, comparison of included with excluded patients, multiple imputation (m = 20) as sensitivity analysis
12d	Methods — statistical methods	If applicable, explain how loss to follow-up was addressed	Section 2.3. Median follow-up reported by reverse Kaplan–Meier (816 days); censoring handled by the Cox and Kaplan–Meier estimators
12e	Methods — statistical methods	Describe any sensitivity analyses	Sections 2.3, 2.6 and 2.8. Progression-free-interval endpoint; median split; multiple imputation; extended-covariate models; spline versus linear; adjustment for cellular composition; permutation null; random-effects meta-analysis across biopsy cohorts
13a	Results — participants	Report numbers of individuals at each stage of study—eg numbers potentially eligible, examined for eligibility, confirmed eligible, included in the study, completing follow-up, and analysed	Section 2.3 and Supplementary Figure S6
13b	Results — participants	Give reasons for non-participation at each stage	Section 2.3. Exclusions: non-primary or duplicate samples, absent *MTARC1* measurement, missing survival, missing covariates
13c	Results — participants	Consider use of a flow diagram	Supplementary Figure S6
14a	Results — descriptive	Give characteristics of study participants (eg demographic, clinical, social) and information on	Section 2.3 and results/11_missing_compare.csv, which gives age, sex, stage, expression and survival for the included and the excluded patients side by side; marker distributions in Figure 2 and results/10_expression_estimates.csv.

#	Item	Recommendation	Where addressed
	data	exposures and potential confounders	No separate table of participant characteristics is presented, so this item is addressed in part
14b	Results — descriptive data	Indicate number of participants with missing data for each variable of interest	results/11_extended_covariate_availability.csv and Section 2.3
14c	Results — descriptive data	Summarise follow-up time (eg, average and total amount)	Section 2.3. Median 816 days (26.8 months)
15	Results — outcome data	Report numbers of outcome events or summary measures over time	Section 2.3. 114 deaths among 339 patients; 162 progression events; GSE14520 96 deaths among 242
16a	Results — main results	Give unadjusted estimates and, if applicable, confounder-adjusted estimates and their precision (eg, 95% confidence interval). Make clear which confounders were adjusted for and why they were included	Sections 2.3, 2.4, 2.7. Unadjusted pan-cancer estimates; the adjusted primary estimate with its covariate set, and the reason those three confounders were chosen given in Section 4.5; external cohort reported both unadjusted and adjusted
16b	Results — main results	Report category boundaries when continuous variables were categorized	Sections 2.3 and 4.5. Stage I–II versus III–IV; cirrhosis as Ishak 5–6 versus 0–4 (Section 2.3); median split defined at the cohort median
16c	Results — main results	If relevant, consider translating estimates of relative risk into absolute risk for a meaningful time period	Not translated to absolute risk, because the ΔC-index of 0.005 (95% CI −0.015 to +0.037) shows the marker adds essentially nothing to a clinical model, so an absolute-risk presentation would overstate its usefulness. Stated in Section 2.3
17	Results — other analys	Report other analyses done — eg analyses of subgroups and interactions, and	Sections 2.5–2.9 and the corresponding deposited result files

#	Item	Recommendation	Where addressed
	es	sensitivity analyses	
18	Discussion — key results	Summarise key results with reference to study objectives	Section 3.1, which separates findings that survive from those that do not
19	Discussion — limitations	Discuss limitations of the study, taking into account sources of potential bias or imprecision. Discuss both direction and magnitude of any potential bias	Section 3.5, covering effect size and clinical increment, weak correlations and composition confounding, resource non-independence, bulk-tissue composition in the biopsy cohorts with the direction of the expected bias stated, transcript–protein divergence, and pharmacological tractability and on-target safety
20	Discussion — interpretation	Give a cautious overall interpretation of results considering objectives, limitations, multiplicity of analyses, results from similar studies, and other relevant evidence	Sections 3.2–3.4. The tissue-context model is labeled a hypothesis in the text and in the caption of Figure 4; the mARC1 in vivo literature is presented including the studies that contradict the protective result
21	Discussion — generalisability	Discuss the generalisability (external validity) of the study results	Section 3.5. GSE14520 is HBV-related and predominantly Chinese; the biopsy cohorts used different scoring systems; generalization to metabolic HCC is not established
22	Other information — funding	Give the source of funding and the role of the funders for the present study and, if applicable, for the original study on which the present article is based	Funding statement: no external funding. Funding of the originating consortia is described in their primary publications, cited in Sections 2.4, 4.2 and 4.7

Summary of STROBE compliance. Of the 34 numbered items, 32 are addressed, item 14a of those in part (no separate table of participant characteristics) and item 5 in part (the accrual window and the calendar end of follow-up are not recoverable from the harmonized public resources). Item 6b is not applicable (no matching).

Item 16c is deliberately not performed, with the reason stated. Item 10 is answered by declaring that no sample-size calculation was performed rather than by supplying a post-hoc one.
